# Triptolide Reduces Cholesterol Synthesis and Alleviates Neuroinflammation by Inhibiting CD33 in Alzheimer’s Disease Development and Progression

**DOI:** 10.3390/biology15110818

**Published:** 2026-05-22

**Authors:** Yi Yang, Yue Ma, Pu Wang, Pei-Pei Guan

**Affiliations:** 1 College of Life and Health Sciences, Northeastern University, Shenyang 110819, China; 2 College of Pharmacy, Shenzhen Technology University, Shenzhen 518118, China

**Keywords:** Alzheimer’s disease, triptolide, neuroinflammation, cholesterol, CD33

## Abstract

Alzheimer’s disease (AD) is a progressive neurodegenerative disorder, which has recently been found to be closely associated with neuroinflammation. As an anti-inflammatory drug, triptolide (TP), a natural diterpenoid from *Tripterygium wilfordii*, was selected in the current study for treating PS19 (tau^P301S^ transgenic) mice, tauopathy AD mice. In addition, we have previously found that TP had the ability to reduce the level of cholesterol. However, the roles and mechanisms of TP in the above processes are not clear. To this end, we found that elevated cholesterol in serum and brain tissues upregulated the expression of apolipoprotein E (APOE) and sialic acid-binding Ig-like lectin 3 (CD33), leading to the activation of SH2-containing protein tyrosine phosphatase 1 (SHP-1). The activation of SHP-1 inhibits the signaling pathways of Janus kinase 1 (JAK1) and signal transducer and activator of transcription 6 (STAT6), which results in inhibition of the M2 polarization of microglia, which exacerbates neuroinflammation and cognitive decline in high-cholesterol diet (HCD)-fed mice. Conversely, TP treatment significantly inhibited the hepatic sterol regulatory element-binding protein 2 (SREBP2)/3-hydroxy-3-methylglutaryl-coenzyme A reductase (HMGCR) pathway, which reduced the cholesterol levels in the serum and brain. By depressing the levels of cholesterol, the axis of CD33 and SHP-1 was suppressed, which resulted in restoration of the activity of JAK1 and STAT6 pathways, leading to the transition of microglia from the M1 to the M2 phenotype. Of note, these observations demonstrate that TP alleviates the cognitive impairment of PS19 mice via depressing neuroinflammation. Altogether, our results revealed the mechanisms of TP in treating AD via CD33/SHP-1/JAK1/STAT6 pathways in a cholesterol-dependent manner.

## Simple Summary

Triptolide reduces cholesterol levels by inhibiting the hepatic SREBP2/HMGCR pathway. This lowers the expression of CD33 and SHP-1, thereby restoring JAK1/STAT6 signaling. Consequently, microglia switch from the pro-inflammatory M1 to the protective M2 phenotype, alleviating neuroinflammation and cognitive decline in a tauopathy mouse model of AD. These findings reveal a cholesterol-dependent mechanism for triptolide’s therapeutic effects.

## 1. Introduction

Alzheimer’s disease (AD) is pathologically characterized by extracellular aggregation of β-amyloid (Aβ) leading to form β-amyloid plaques (APs) and intracellular hyperphosphorylation of tau protein to neurofibrillary tangles (NFTs) [1,2]. Familial early-onset AD is caused by mutations in amyloid precursor protein (APP) and presenilin 1 (PS1). In contrast, tauopathies including the PS19 mouse model used in this study are characterized primarily by tau hyperphosphorylation and aggregation [1]. The APP/PS1 (B6.Cg-Tg(APPswe,PSEN1dE9)85Dbo/Mmjax) double transgenic (Tg) mouse is a classic model of AD characterized by robust AP pathology. The PS19 mouse, on the other hand, overexpresses the human tau^P301S^ mutation and recapitulates key features of tauopathy, including tau-dependent neurodegeneration and cognitive decline [2]. Both Aβ and tau pathologies trigger chronic neuroinflammation, which further accelerates synaptic damage and neuronal loss [3,4].

Recent studies have revealed that metabolic disorders and immune dysregulation are two core mechanisms driving the onset and progression of AD. These two mechanisms are interconnected through the common target of “neuroinflammation”, forming a complex pathological regulatory network. From a metabolic perspective, AD exhibits significant “metabolic disease” characteristics. It shares multiple pathological mechanisms with type 2 diabetes, such as insulin resistance and abnormal glucose–lipid metabolism [5]. Among various metabolic abnormalities, the association between cholesterol metabolism disorders and AD is particularly prominent: epidemiological studies have confirmed high cholesterol as an independent risk factor for AD. Moreover, diet-induced hypercholesterolemia directly causes cognitive impairment in mice [6]. Clinical data further demonstrate that serum cholesterol concentrations in AD patients gradually rise as the disease progresses [7]. Further mechanistic studies indicate that abnormally elevated cholesterol not only promotes the aggregation of Aβ in the brain to form APs but also impairs the integrity of the blood–brain barrier (BBB) [8]. More importantly, it can directly activate oxidative stress responses in the central nervous system (CNS), thereby inducing excessive activation of microglia and ultimately exacerbating neuroinflammatory damage [9]. This suggests that cholesterol may act as a key bridge connecting peripheral metabolic abnormalities to central pathological damage by regulating neuroinflammation.

A series of studies indicate that immune system dysfunction is closely linked to the onset of AD [10]. Microglia are the principal immune cells in the CNS that identify and eliminate detrimental chemicals from the brain [11]. During AD progression, microglia are activated to engulf and remove harmful substances such as Aβ, thereby maintaining brain homeostasis [12]. In addition to clearing out harmful substances, the immune system is also involved in repairing the impaired brain [13]. Specifically, microglia and other immune cells promote neuronal regeneration [14]. At the same time, activated microglia could secrete a number of cytokines and chemicals, which exert effects of neuroprotection and neurotoxicity [15]. Among the molecules that regulate microglial activation, sialic acid-binding Ig-like lectin 3 (CD33) plays a crucial role. CD33 is mainly expressed in myeloid cells, including microglia, and its expression level is significantly positively correlated with both Aβ deposition in the brain and the degree of neuroinflammation. Conversely, inhibition of CD33 effectively reduces pro-inflammatory microglial activation and decreases Aβ aggregation [16].

Notably, although cholesterol and CD33 belong to different regulatory systems, both target neuroinflammation, which is the core pathological link of AD. Whether there is a direct regulatory association between them remains a key unresolved scientific question in current research on the metabolic–immune mechanisms of AD. Answering this question will provide core clues for understanding “how metabolic abnormalities drive AD progression through immune pathways” and lay a theoretical foundation for the development of AD intervention strategies targeting both metabolism and immunity.

Triptolide (TP), a natural diterpenoid derived from the traditional Chinese herb *Tripterygium wilfordii*, has emerged as a focal point of investigation among prospective treatment agents for AD due to its notable anti-inflammatory properties and neuroprotective benefits. Previous studies have shown that TP exerts anti-inflammatory effects primarily by inhibiting nuclear factor kappa-light-chain-enhancer of activated B cells (NF-κB) signaling and reducing proinflammatory cytokines such as interleukin (IL)-1β and IL-6, a mechanism widely exploited in autoimmune diseases [17]. In addition, the role of TP has been further exploited in the field of AD. For example, TP can alleviate AD pathology by activating the nuclear factor erythroid 2-related factor 2 (Nrf2) pathway and inhibiting the NF-κB pathway [18]. Furthermore, TP can also specifically inhibit the excessive activation of astrocytes and microglia in the brains of APP/PS1 transgenic mice, while reducing the production and deposition of Aβ [19,20,21].

Of note, recent studies have found that TP has the potential ability to regulate cholesterol metabolism. In AD mice, TP reduces serum cholesterol levels by inhibiting AMP-activated protein kinase (AMPK), a key regulator of lipid and cholesterol metabolism [22,23]. Considering the known roles of cholesterol and CD33 in neuroinflammation, along with the dual properties of TP, a key hypothesis arises that TP may regulate cholesterol metabolism to modulate CD33 expression or activity, thus reducing microglia-driven neuroinflammation and ameliorating AD pathology.

However, the molecular links by which TP regulates cholesterol metabolism, its impact on CD33-mediated neuroinflammation, and the role of the “TP-cholesterol-CD33” pathway in AD remain uncharacterized. In view of these problems, the present study aims to verify the following core hypothesis that TP reduces cholesterol levels, leading to downregulation of CD33 expression and activity and inhibition of microglial overactivation as well as the release of proinflammatory factors, which result in an alleviation of cognitive impairment and AD pathology. This study provides new insights into the metabolic–immune regulatory network in AD and establishes a foundation for TP-based therapeutic strategies.

## 2. Materials and Methods

### 2.1. Reagents

An antibody against CD33 (bs-1514R, rabbit, 1:1000 for western blot) was obtained from BiossAntibody (Beijing, China). Antibodies against allograft inflammatory factor-1 (Iba1) (#17198, rabbit, 1:2000 for western blot, 1:200 for IHC), IL-1β (#12242, mouse, 1:2000 for western blot), apolipoprotein E (APOE) (#49285, rabbit, 1:2000 for western blot), arginase-1 (Arg1) (#43933, mouse, 1:2000 for western blot), SH2-containing protein tyrosine phos-phatase-1 (SHP-1) (#26516, rabbit, 1:1000 for western blot), and β-actin (#3700, mouse, 1:5000 for western blot) were obtained from Cell Signaling Technology (Shanghai, China). Antibodies against sterol regulatory element-binding protein 2 (SREBP2) (YP-Ab-04920, rabbit, 1:2000 for western blot), 3-hydroxy-3-methylglutaryl-coenzyme A reductase (HMGCR) (YP-Ab-17137, rabbit, 1:2000 for western blot), Janus kinase 1 (JAK1) (YP-Ab-14799, rabbit, 1:2000 for western blot), p-JAK1 (YP-Ab-14595, rabbit, 1:2000 for western blot), signal transducer and activator of transcription 6 (STAT6) (YP-Ab-01024, rabbit, 1:2000 for western blot) and p-STAT6 (YP-Ab-01284, rabbit, 1:2000 for western blot) were obtained from Upingbio (Shenzhen, China). All reagents for the sodium dodecyl sulfate polyacrylamide gel electrophoresis (SDS-PAGE) experiments and horseradish peroxidase (HRP)-labeled secondary antibodies were purchased from Servicebio (Wuhan, China). The cholesterol detection kit was purchased from Rayto (Shenzhen, China) which determines the levels of cholesterol according to the cholesterol oxidase–peroxidase aminophenazone (CHOD-PAP) method.

### 2.2. Tg Mice and Treatment

PS19 mice (tau^P301S^ transgenic; Cavens (Changzhou, China), stock No. 024841) were used as an AD model, with C57BL/6 mice as wild-type (WT) controls. Genotyping analysis was performed after the mice were one month of age. Mice weighing 18–20 g were randomly assigned to experimental groups. They were housed under SPF conditions (3–4 per cage) with standard bedding and nesting material, at 23 ± 2 °C, 50–60% humidity, and a 12 h light/dark cycle. Beginning at 4 mon of age, the mice were administered either a standard diet or a high-cholesterol diet (HCD) for 8 weeks with high-cholesterol feed (1.25% cholesterol and 0.5% sodium cholate, obtained from Trophic (Nantong, China)). PS19 mice in the TP treatment group were further randomized into subgroups. At 4 mon of age, the PS19 mice were administered TP intranasally at a dosage of 20 μg/kg every 2 d for a duration of 5 mon [22]. Cognitive function was assessed using the Morris water maze and novel object recognition (NOR) tests. After behavioral testing, mice were sacrificed by intraperitoneal injection of sodium pentobarbital solution with the dose of 200 mg/kg body weight, after which the mice will be perfused, and the brains and livers will be collected for the subsequent analyses.

### 2.3. Morris Water Maze Test

In this study, a Morris water maze was employed to assess spatial learning and memory in mice. The apparatus consisted of a white circular pool (120 cm in diameter, 45 cm in height) and a fixed white circular platform (10 cm in diameter). The water temperature was consistently maintained at 22 ± 0.5 °C throughout the experiment. The pool was virtually divided into four equal quadrants, and the platform was always positioned at the center of the target quadrant. The experimental procedure lasted 8 consecutive days. On day 1, a visible platform trial was performed to acclimate the mice to the environment. From days 2 to 7, hidden platform training was performed, with the platform submerged 0.5 cm below the water surface. On day 8, a spatial probe trial was conducted with the platform removed. During training, each mouse underwent four trials per day, starting from random entry points in different quadrants. The mice were gently placed into the water facing the pool wall, and each trial had a maximum duration of 60 s. If a mouse failed to locate the platform within 60 s, it was gently guided onto the platform and allowed to remain for 15 s. In the probe trial, mice were allowed to swim freely for 60 s, and their behavior was recorded. Throughout the experiment, an automated video tracking system was used to record swimming trajectories. The system automatically analyzed the following parameters: escape latency (time taken to reach the platform) and the number of crossings over the original platform location.

### 2.4. Novel Object Recognition Test (NOR)

Mice were placed in an open box (40 × 40 × 40 cm) facing away from two identical objects. Mice were allowed to explore the objects freely for 5 min, then returned to their home cage for a 1 h retention period. To assess recognition memory, one object was replaced with a novel object. Mice were then returned to the box and allowed to explore freely for an additional 5 min (test phase). The number and duration of object explorations were recorded during both phases.

The recognition index = the number of times touching the new item/the number of times touching both items.

### 2.5. Cell Culture

Mouse microglial BV2 and liver-derived alpha mouse liver 12 (AML-12) cells were cultured in 6 cm dishes in Dulbecco’s modified eagle medium (DMEM) (Servicebio, Wuhan, China) containing 10% fetal bovine serum (FBS) (Abcell, Beijing, China) at 37 °C with 5% CO_2_. In an independent experimental cohort, BV2 cells were pre-incubated in serum-free medium for 24 h, followed by exposure to cholesterol with a concentration gradient of 0–200 μM. Mouse liver-derived AML-12 cells were cultured in DMEM with 10% FBS under the same conditions. A 12 h serum-free adaptation period was implemented for AML-12 cells before they were treated with 50 nM TP.

### 2.6. siRNA Transfection

The small interfering (si) RNA targeting APOE, CD33 or SHP-1 was synthesized by Sangon (Shanghai, China), with a negative siRNA used as the control. Transfection was performed using Lipofectamine 8000 (Beyotime, Beijing, China). The sequences of siRNA were as follows: siRNA-CD33, AGAGCAGUCCACACUAA; siRNA-APOE, GGAGGACACUAUGACGGAA; siRNA-SHP-1, GACUAUUCUGUAUGCACUU; and siRNA-control, UUCUCCGAACGUGUCACGU.

### 2.7. Western Blot (WB)

Tissue samples were homogenized in ice-cold RIPA lysis buffer supplemented with a protease inhibitor cocktail (1:100, Beyotime, Beijing, China), 1 mM sodium fluoride (NaF), and 1 mM sodium orthovanadate (Na_2_VO_4_). After centrifugation at 12,000× *g* for 10 min at 4 °C, protein concentrations were determined using the bicinchoninic acid (BCA) Protein Assay Kit (Beyotime, Beijing, China). Equal quantities of protein were resolved by SDS-PAGE on 10% Bis-Tris gels utilizing Tris–Glycine running buffer, followed by transfer onto polyvinylidene difluoride (PVDF) membranes. Membranes were blocked with 5% skim milk, incubated with primary antibodies overnight at 4 °C, then with HRP-conjugated secondary antibodies for 1 h at room temperature. Immunoreactive bands were detected using an enhanced chemiluminescence detection kit (Tanon, Shanghai, China) and photographed with a Tanon 5500 Chemiluminescence Imaging System. Densitometric measurement of immunoreactive signals was conducted using ImageJ (1.49m, NIH).

### 2.8. Immunohistochemistry (IHC)

The brains of the mice were excised and immediately fixed in 4% paraformaldehyde prior to paraffin embedding. Tissue slices were sliced to a thickness of 10 μm and affixed to Superfrost Plus slides (VWR Scientific, West Chester, PA, USA). Following deparaffinization in xylene and rehydration through a graded ethanol series, the sections were rinsed in phosphate-buffered saline (PBS, pH 7.4). Endogenous peroxidase activity was quenched by incubation with 3% hydrogen peroxide for 15 min. The sections were then incubated overnight at 4 °C with a rabbit polyclonal antibody against Iba1. After washing, the slides were treated with a biotin-conjugated secondary antibody for 1 h at room temperature, followed by incubation with streptavidin-HRP for 30 min. Immunoreactivity was visualized using 3,3′-diaminobenzidine (DAB) as the chromogen, applied for 1 min, and the reaction was terminated by washing in distilled water for 10 min. Sections were lightly counterstained with hematoxylin for 30 s, differentiated briefly in acid alcohol, and then dehydrated and mounted. Images were captured using a Leica microscope (CM1850, Leica, Wetzlar, Germany).

### 2.9. Statistical Analysis

All data are presented as the means ± standard error of the mean (S.E.M.) of at least three independent experiments. The normality of the distribution was assessed using the Shapiro–Wilk test. Statistical analysis was performed using GraphPad Prism 10.4.1 software. The statistical significance of the differences between the means was determined using Student’s *t* test for two independent groups or one-way analysis of variance (ANOVA) for multiple groups, where appropriate. If the means were significantly different, multiple pairwise comparisons were performed using Tukey’s post hoc test (* *p* < 0.05, ** *p* < 0.01, *** *p* < 0.001).

## 3. Results

### 3.1. CD33 Is Induced in 9 Mon Old PS19 Mice, Which Potentially Contributes to Neuroinflammation and Cognitive Decline

**Figure 1:**
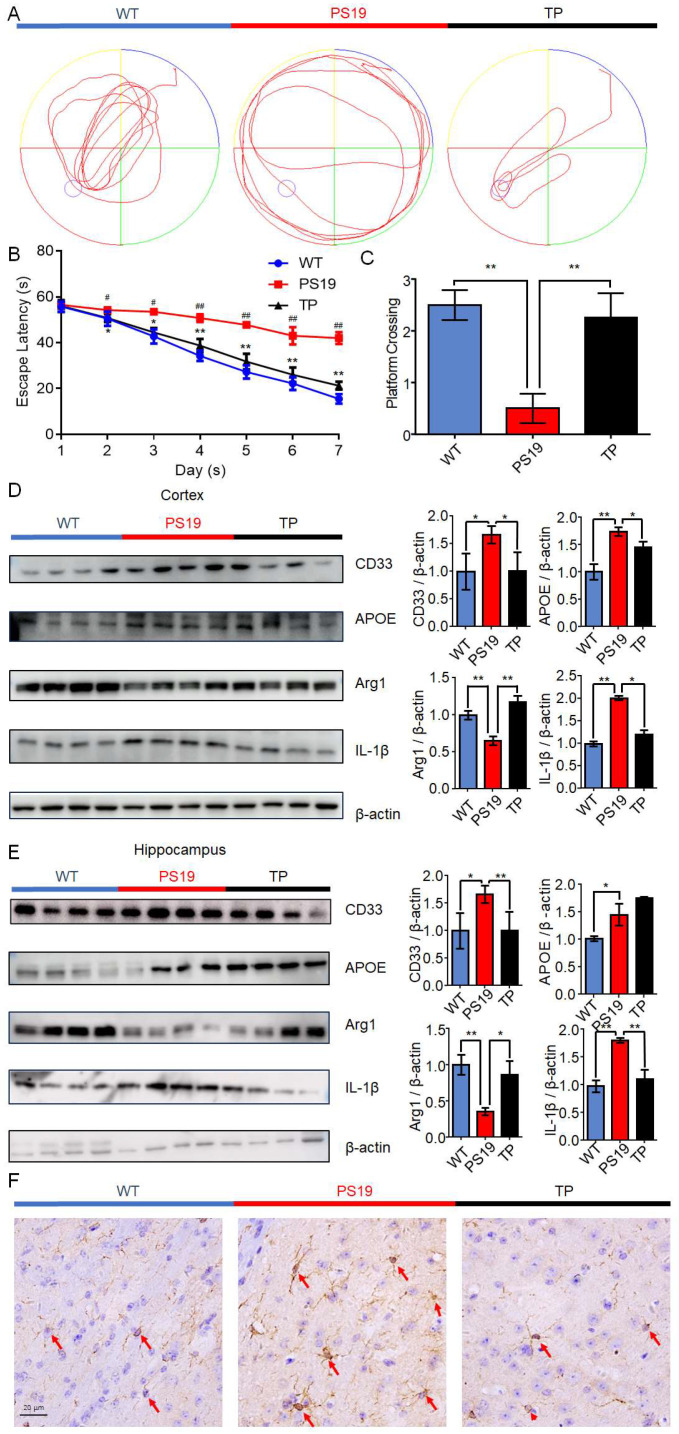
TP treatment inhibits neuroinflammation via reducing the expression of CD33, which results in alleviating cognitive impairment. PS19 mice at the age of 4 mon old were treated with TP for 5 mon. (**A**) Representative images of swim paths were shown in the probe trial of the Morris water maze tests. (**B**) Escape latency was measured in the training trial tests. (**C**) The number of platform crossings was recorded in the probe trial tests. (**D**,**E**) Western blot analysis was used to detect the expression of CD33, APOE, IL-1β and Arg1 in the cortex and hippocampus of WT, PS19, and PS19 mice treated with TP (*n* = 5). The optical density of the bands was analyzed by ImageJ. β-actin served as an internal control. The protein expression level was expressed as a fold change relative to that of WT mice, which was set to 1. (**F**) Iba1 was immunostained in the cortex of the PS19 mice by IHC, arrows point to immunostained BV2 cells. The data are presented as the mean ± S.E.M. of independent experiments. * *p* < 0.05; ** *p* < 0.01; compared with PS19 mice. # *p* < 0.05; ## *p* < 0.01; compared with WT mice. Original images are available in the Supplementary Materials.

To determine the relationship between CD33 and AD, we first characterized CD33 expression and related pathological changes in PS19 mice, a tauopathy model of AD. Compared with WT mice, PS19 mice showed cognitive impairment in the Morris water maze (Figure 1A–C) and NOR tests [22]. We next assessed the protein expression of CD33 and the associated neuroinflammatory factors in 9 mon old PS19 mice. The protein expression of CD33, APOE, and IL-1β were elevated in PS19 mice compared with that of WT mice (Figure 1D,E). The expression of Arg1, a hallmark of M2 polarization in microglia, was decreased in the brains of PS19 mice (Figure 1D,E). To further determine the activity of microglia, IHC assays were carried out to immunostain Iba1 in WT and PS19 mice. The results demonstrated that microglia were activated in PS19 mice compared to those in WT mice (Figure 1F). These results demonstrate that elevated CD33 expression is closely associated with microglia activation and cognitive impairment in AD.

### 3.2. Triptolide Reduces the Level of CD33, Through Which It Inhibits Neuroinflammation in PS19 Mice

As the above results show, the levels of CD33 and neuroinflammatory factors were enhanced in PS19 mice, and we next investigated whether TP mitigates neuroinflammation in AD. Western blot analysis revealed that TP reduced the protein levels of CD33, APOE, and IL-1β in the cerebral cortex and hippocampus of PS19 mice (Figure 1D,E). In addition, IHC tests were conducted to evaluate the morphology of microglia by immunostaining Iba1. The results demonstrated that TP inhibits microglial activation in PS19 mice (Figure 1F). Given these observations, Morris water maze tests were carried out to determine the behavior of different groups of mice. The results demonstrated that TP inhibited the cognitive decline of PS19 mice (Figure 1A–C). Based on these observations, TP showed its ability to inhibit neuroinflammation and ameliorate cognitive decline in tauopathy mice.

### 3.3. High-Cholesterol Diet Increases CD33 Expression in Brain, Inducing Neuroinflammation and Cognitive Impairment

**Figure 2:**
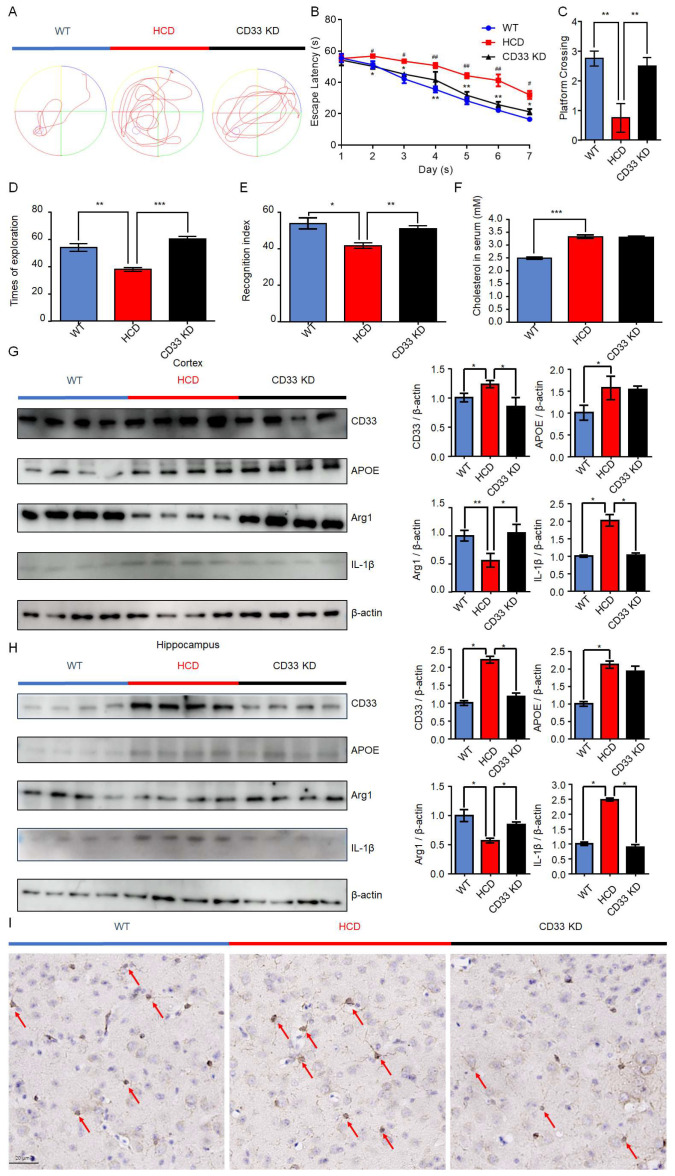
High-cholesterol diet induces neuroinflammation and cognitive impairment, which was partially reversed by knocking down the expression of CD33. C57BL/6 mice were treated with HCD in the absence or presence of AAV-shRNA-CD33. (**A**) Representative images of swim paths were shown in the probe trial of the Morris water maze tests. (**B**) Escape latency was measured in the training trial tests. (**C**) The number of platform crossings was recorded in the probe trial tests. (**D**) The number of exploration times were determined by the NOR tests. (**E**) The recognition index was determined by the NOR tests. (**F**) The concentration of cholesterol in serum was determined by the CHOD-PAP tests. (**G**,**H**) Western blot analysis was used to detect the expression of CD33, APOE, IL-1β and Arg1 in the cortex and hippocampus of WT mice, which were treated with HCD in the absence or presence of AAV-shRNA-CD33 (*n* = 4). The optical density of the bands was analyzed by ImageJ. β-actin served as an internal control. The protein expression level was expressed as a fold change relative to that of WT mice, which was set to 1. (**I**) Iba1 was immunostained in the cortex of the WT HCD and HCD treated with AAV-shRNA-CD33 mice by IHC, arrows point to immunostained BV2 cells. The data are presented as the mean ± S.E.M. of independent experiments. * *p* < 0.05; ** *p* < 0.01; *** *p* < 0.001; compared with HCD mice. # *p* < 0.05; ## *p* < 0.01; compared with WT mice. Original images are available in the Supplementary Materials.

To investigate the relationship between serum cholesterol, CD33, neuroinflammation and cognitive impairment, WT mice were subjected to treatment with HCD for 8 weeks in the absence or presence of CD33 knockdown. Compared to WT mice, HCD-treated mice showed a cognitive impairment in the Morris water maze and NOR tests, with an elevated level of cholesterol in serum (Figure 2A–F). CD33 knockdown ameliorated the HCD-induced cognitive impairment without reducing serum cholesterol levels (Figure 2A–F). Concurrently, HCD upregulated the expression of CD33, APOE and IL-1β, whereas it downregulated the expression of Arg1 in C57BL/6 mice (Figure 2G,H). These effects were reversed by knocking down the expression of CD33, except for APOE (Figure 2G,H). Furthermore, the IHC results demonstrated that CD33 knockdown deactivates the microglia of HCD-treated mice (Figure 2I). Therefore, these findings suggest that HCD promotes neuroinflammation and cognitive impairment, which are partially mitigated via CD33 knockdown.

**Figure 3:**
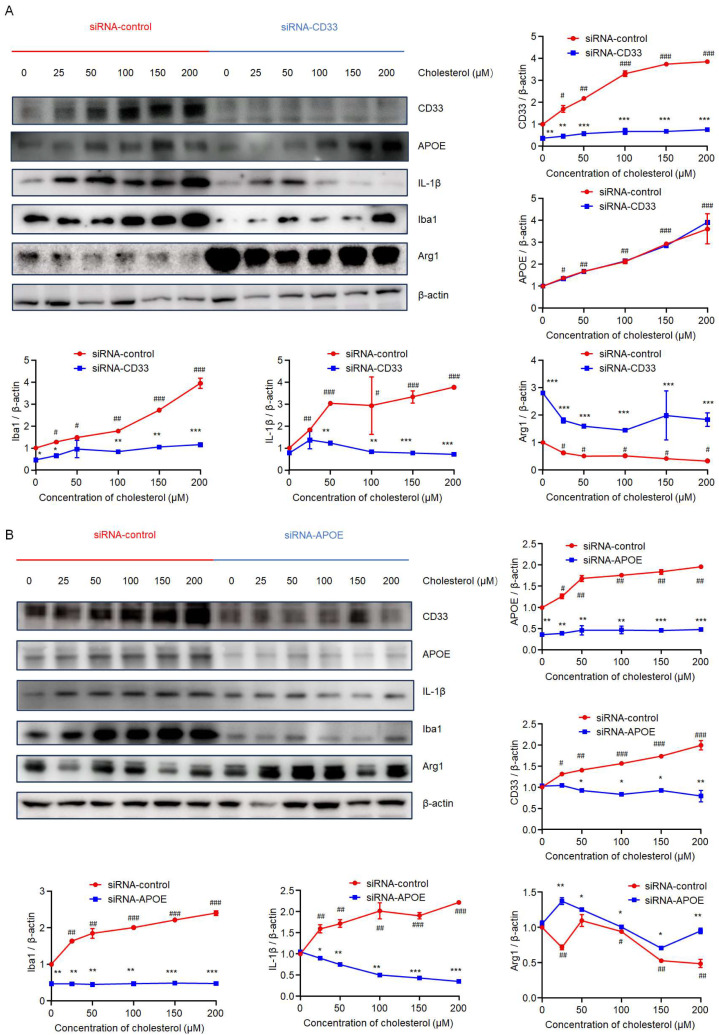
Knocking down the expression of CD33 or APOE blocked the effects of cholesterol on inducing neuroinflammation. (**A**) BV2 cells were transfected with siRNA-CD33 or siRNA-control before being treated with the indicated concentration of cholesterol ranging from 0 to 200 μM. After 24 h of treatment, western blot experiments were carried out to determine the expression of CD33, APOE, Iba1, IL-1β and Arg1. (**B**) BV2 cells were transfected with siRNA-APOE or siRNA-control before being treated with the indicated concentration of cholesterol ranging from 0 to 200 μM. After 24 h, western blot experiments were carried out to determine the expression of CD33, APOE, Iba1, IL-1β and Arg1. The optical density of the bands was analyzed by ImageJ. β-actin served as an internal control. The protein expression level was expressed as a fold change relative to that of the BV2 cells transfected by siRNA-control in the absence of cholesterol, which was set to 1. The data are presented as the mean ± S.E.M. of independent experiments. * *p* < 0.05; ** *p* < 0.01; *** *p* < 0.001; compared with the BV2 cells transfected by siRNA-control, # *p* < 0.05; ## *p* < 0.01; ### *p* < 0.001; compared with the BV2 cells transfected by siRNA-control in the absence of cholesterol. Original images are available in the Supplementary Materials.

In view of the above results, in vitro experiments were carried out to evaluate the effects of cholesterol on the expression of CD33 and inflammatory factors. For this purpose, we treated the BV2 cells with the indicated concentration of cholesterol ranging from 0 to 200 μM. After 12 h of treatment, we found that cholesterol increased the protein expression of CD33, APOE, Iba1 and IL-1β, and decreased the level of Arg1 (Figure 3A). In addition, we compared the protein expression of CD33, APOE, IL-1β, Iba1 and Arg1 in the absence of cholesterol treatment. The results demonstrated that CD33 knockdown concurrently decreased the expression of Iba1 and increased the expression of Arg1 in BV2 cells (Figure 3A). After addition of cholesterol, we further found that CD33 siRNA transfection reduced the expression of Iba1 and IL-1β and increased the expression of Arg1 in cholesterol-treated BV2 cells (Figure 3A).

To continue investigating the relationship between CD33 and APOE, we knocked down the expression of APOE by siRNA in BV2 cells. By western blot, we found that knocking down the expression of APOE blocked the effects of cholesterol on inducing the expression of CD33 (Figure 3B). These results indicate that CD33 is located downstream of APOE to regulate the expression of Iba1, IL-1β and Arg1. Furthermore, APOE knockdown also ameliorated the neuroinflammation via decreasing the expression of Iba1 and IL-1β as well as restoring the expression of Arg1 (Figure 3B). When we solely investigated the roles of APOE in the above genes, we did not find its effects on most of the proteins (Figure 3B), which might be caused by the absence of cholesterol. All these results revealed that CD33 plays key roles in mediating the effects of cholesterol on neuroinflammation.

### 3.4. Triptolide Treatment Affects the Metabolisms of Cholesterol Through the SREBP2/HMGCR Pathway in the Liver, but Not in the Brain

**Figure 4:**
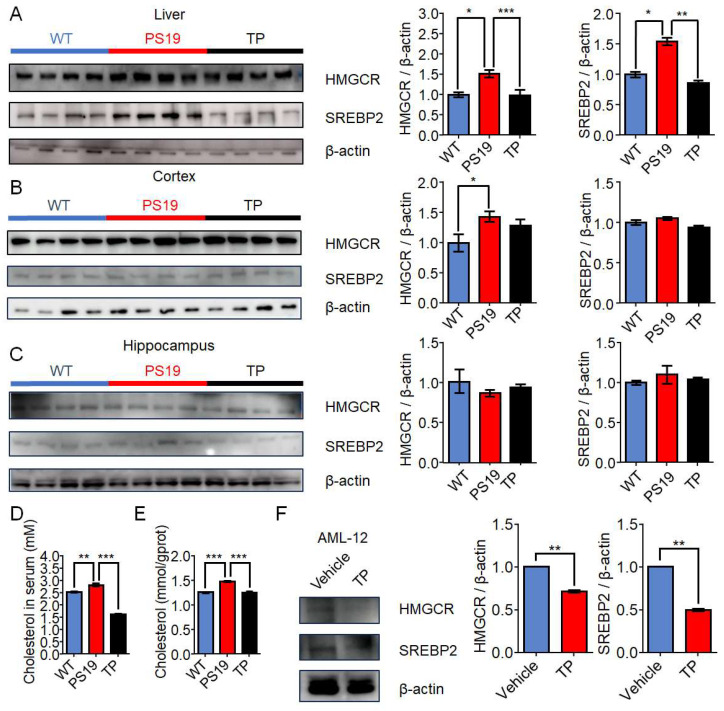
TP reduces the levels of cholesterol in serum via the SREBP2/HMGCR pathway in liver, which results in similar alternations in the brain. PS19 mice at the age of 4 mon old were treated with TP for 5 mon. (**A**–**C**) Western blot analysis was used to detect the expression of HMGCR and SREBP2 in the liver, the cortex and hippocampus of different groups of mice (*n* = 5). The levels of cholesterol in serum (**D**) or brains (**E**) were determined by the CHOD-PAP tests. (**F**) Western blot analysis was used to detect the protein levels of HMGCR and SREBP2 in AML-12 cells treated with TP. The optical density of the bands was analyzed by ImageJ. β-actin served as an internal control. The protein expression level was expressed as a fold change relative to that of WT mice or vehicle-treated AML-12 cells, which was set to 1. The data are presented as the mean ± S.E.M. of independent experiments. * *p* < 0.05; ** *p* < 0.01; *** *p* < 0.001; compared with PS19 mice or vehicle-treated AML-12 cells. Original images are available in the Supplementary Materials.

HMGCR, a crucial enzyme in cholesterol biosynthesis, functions as the rate-limiting enzyme for endogenous cholesterol synthesis. We thereby aimed to examine the effects of TP on modulating the expression of HMGCR and its transcriptional factor SREBP2, leading to the synthesis of cholesterol. As expected, the protein expression of HMGCR and SREBP2 were increased in the livers of PS19 mice. More interestingly, TP treatment partially restores the protein levels of HMGCR and SREBP2 in the hepatic tissues of PS19 mice (Figure 4A). Nonetheless, only the protein expression of HMGCR was increased in PS19 mice, and TP is unable to diminish the protein levels of HMGCR and SREBP2 in the cerebral cortex and hippocampus of PS19 mice (Figure 4B,C). In addition, PS19 mice showed relatively higher levels of cholesterol in the serum, which was reduced by the addition of TP (Figure 4D). Likewise, the levels of cholesterol in the brains of PS19 mice were also increased compared to those of WT mice, which were decreased by the addition of TP (Figure 4E). Furthermore, in vitro research utilizing AML-12 liver cells corroborated that TP diminishes the protein levels of HMGCR and SREBP2 (Figure 4F). Taken together, these data demonstrate that TP suppresses the synthesis of cholesterol in the liver, which influences the levels of cholesterol in the brain.

### 3.5. Triptolide Restores the Activities of JAK1 and STAT6 Pathways in CD33- and SHP-1-Dependent Mechanisms, Which Results in Reduced Expression of Arg1

**Figure 5:**
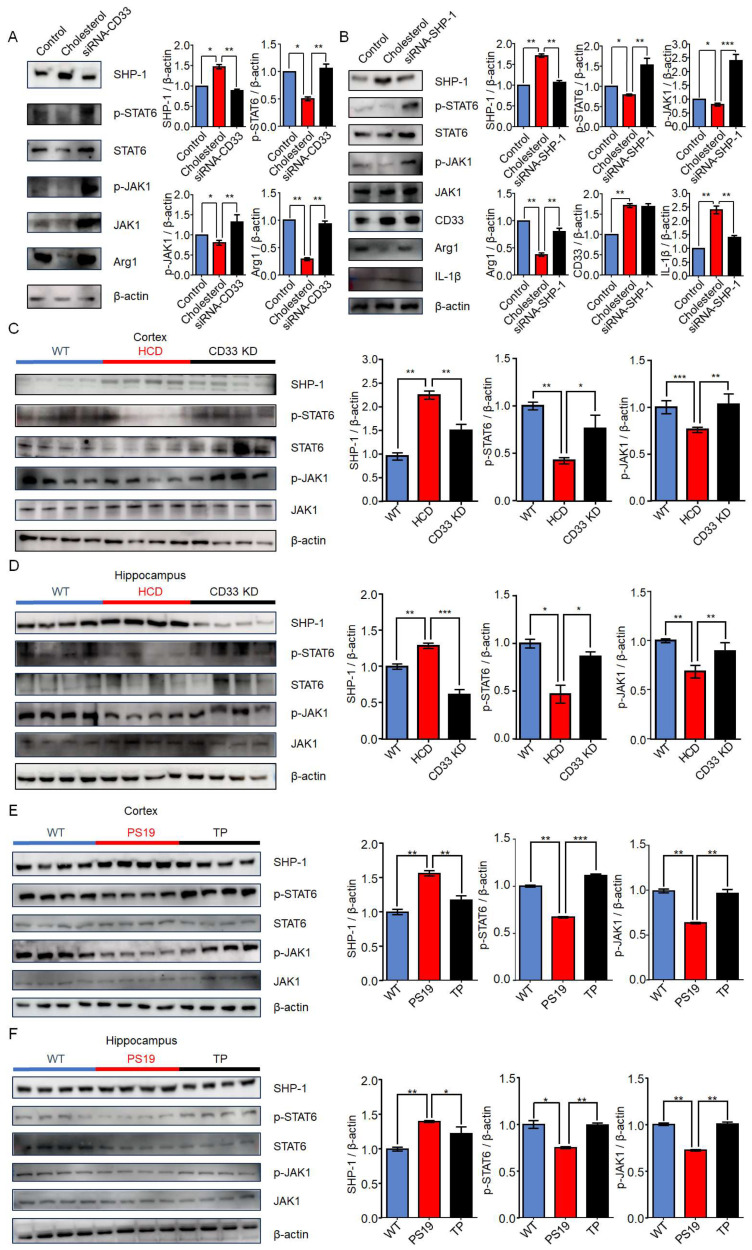
TP activates the JAK1/STAT6 pathway via inhibiting SHP-1, which is responsible for blocking the effects of cholesterol on inducing neuroinflammation. BV2 cells were treated with cholesterol in the absence or presence of siRNA-CD33 (**A**) or siRNA-SHP-1 (**B**). Western blot analysis was carried out to determine the protein expression of CD33, SHP-1, JAK1, STAT6 and IL-1β as well as the phosphorylation of JAK1 and STAT6. (**C**,**D**) C57BL/6 mice were treated with HCD in the absence or presence of AAV-shRNA-CD33 (*n* = 4). Western blot analysis was carried out to determine the protein expression of SHP-1, JAK1 and STAT6 as well as the phosphorylation of JAK1 and STAT6. (**E**,**F**) PS19 mice were treated with TP (*n* = 5). Western blot analysis was carried out to determine the protein expression of SHP-1, JAK1 and STAT6 as well as the phosphorylation of JAK1 and STAT6. The optical density of the bands was analyzed by ImageJ. β-actin served as an internal control. The protein expression level was expressed as a fold change relative to that of WT mice or BV2 cells transfected by siRNA-control, which was set to 1. The data are presented as the mean ± S.E.M. of independent experiments. * *p* < 0.05; ** *p* < 0.01; *** *p* < 0.001; compared with the BV2 cells transfected by control siRNA, HCD mice or PS19 mice. Original images are available in the Supplementary Materials.

We found that CD33 knockdown reduced SHP-1 expression (Figure 5A), whereas SHP-1 knockdown did not affect CD33 (Figure 5B), indicating that CD33 is located upstream of SHP-1. Given the fact that SHP-1 dephosphorylates JAK kinases [24,25,26], we continued to determine the activities of JAK1/STAT6 pathways. Using western blot, we found that cholesterol suppressed the activities of JAK1/STAT6 signaling pathways, which was reversed by knocking down the expression of CD33 (Figure 5A) or SHP-1 (Figure 5B). To further determine the mechanism of CD33 in regulating neuroinflammation, we further treated the BV2 cells with cholesterol after knocking down the expression of CD33 or SHP-1. By western blot, we found that CD33 silencing abrogated cholesterol-induced SHP-1 expression and reduced the phosphorylation of JAK1 and STAT6 as well as the expression of Arg1 (Figure 5A). Similarly, SHP-1 knockdown also blocked the effects of cholesterol on deactivating JAK1/STAT6 and Arg1 (Figure 5B). Similar results were obtained in vivo (Figure 5C,D). Given the above mechanisms, we further investigated whether TP exerts its anti-inflammatory effects via similar mechanisms. The results demonstrated that TP treatment decreased the expression of SHP-1, while enhancing the phosphorylation of JAK1 and STAT6 as well as the expression of Arg1 in the cerebral cortex and hippocampus of PS19 animals (Figure 5E,F). Therefore, TP exerts its anti-inflammatory effects via SHP-1/JAK1/STAT6 signaling pathways.

## 4. Discussion

As previous studies have revealed the close association between circulating cholesterol metabolism dysregulation and the onset and progression of AD, we thereby extended the prior works to novelly identify the critical mediating role of CD33-expressing innate immune cells in circulating cholesterol-driven neuroinflammation and AD pathological progression. Since the underlying immune mechanisms by which peripheral metabolic abnormalities influence central pathological damage in AD remain largely unclear, our findings systematically validated the cholesterol–CD33 axis as a core bridge linking peripheral cholesterol metabolism disorder to neuroinflammation and cognitive impairment in AD, both in in vivo mouse models and in vitro cell models. These results highlight the complex interplay between systemic metabolic processes and central innate immune homeostasis in the context of AD, which provides potential metabolic and immune dual-targeting strategies for combating AD.

Investigating the mediating effects of immune cells in metabolic abnormality-driven AD requires an understanding of the possible pathways by which these immune cells affect AD risk. CD33, a myeloid cell-specific surface receptor, is the key immune phenotype and molecular target emphasized in this study. CD33 is predominantly expressed in myeloid lineage immune cells including brain-resident microglia and peripheral monocytes/macrophages. These cells have different functions in immune responses and are connected to the pathophysiology of AD through a number of pathways. Notably, in AD, peripheral immune cells can enter cerebral blood vessels and parenchyma, where they aid in brain immunity, the removal of harmful proteins, and regulating neuroinflammation [27]. Peripheral immunity can affect AD in three ways: first of all, peripheral immune cells can bypass the BBB through the brain choroid plexus [28] or through special skull bone marrow channels [29] to enter the brain. Secondly, peripheral immune cells may infiltrate the brain through immune infiltration or recruitment [30]. For example, monocytes can be recruited by CCL2, through which they enter the CNS [31]. Most importantly, there are certain inflammatory molecular interactions between peripheral immunity and the CNS. For example, in the case of high levels of peripheral inflammation, the clearance of Aβ by microglia was markedly impaired in the brains of APP/PS1 mice [32]. Regarding the mechanisms, CD33 might participate in clearing Aβ as a sialic-binding receptor expressed in both myeloid and microglial cells [33]. In addition, the upregulation of CD33 is associated with the deposition of Aβ, leading to neuroinflammation, suggesting its role in the clearance of Aβ [16]. In agreement with the above studies, inhibition of CD33 can reduce the accumulation of Aβ and associated neuroinflammation by a microRNA. Bone marrow dendritic cells are key antigen-presenting cells. They play a crucial role in initiating and regulating adaptive immune responses. During the course of AD, these cells influence disease progression by regulating T cell responses to Aβ. This regulatory function helps clear Aβ. The interaction between dendritic cells and circulating metabolites can affect their maturation and antigen presentation ability, thereby indirectly affecting the risks of AD [34]. In short, the role of immune cells in AD embodies the complex interplay of the immune system and metabolic factors during the course of AD development and progression. These cells act as mediators between metabolic alterations and AD by responding to circulating metabolites. Furthermore, the activation of these immune cells might influence inflammation, which is relevant to the pathogenesis of AD. This understanding highlights the research potential for immune cells that could be influenced by metabolic factors, leading to the risks of AD.

Total cholesterol in serum includes LDL, high-density lipoprotein (HDL), and very low-density lipoprotein (VLDL) cholesterol. For cholesterol, it is associated with neurodegenerative diseases, including AD [35]. Some studies have pointed out that high levels of cholesterol are a risk factor for AD. For instance, diet-induced hypercholesterolemia has the ability to impair the cognitive ability of mice [6]. In metabolomic and epidemiological studies, the average concentration of serum cholesterol has increased during the course of AD development and progression [36,37]. Elevated levels of cholesterol may induce the formation of β-amyloid plaques (APs) and affect their transportation and clearance, but it is still not clear if the cholesterol in serum is related to the production of Aβ or phosphorylation of tau [38]. Furthermore, a study has shown that high levels of cholesterol in blood could damage the BBB and increase the concentration of cholesterol in the rat brain [8]. In line with these observations, excess loading of cholesterol can induce oxidative stress and inflammation. This phenomenon is particularly severe in people with excess loading of LDL. These processes are thought to be responsible for the neurodegenerative changes observed in AD [9]. Alterations in the levels of cholesterol can affect the production and clearance of Aβ, leading to influencing the progression of AD. In addition, deficiency in cellular cholesterol trafficking enhances the generation of Aβ and hyperphosphorylation of tau [39]. Moreover, elevated LDL is involved in increasing the production of Aβ in the CNS [40]. In addition, LDL cholesterol is confirmed as a risk factor of AD, especially in people younger than 65 years [41]. In our study, we found that cholesterol and LDL can increase the risk of AD through specific immune cell types. Other mediators or additional research methods can be considered to further clarify the relationship between cholesterol and AD.

In this study, mutations at CD33 and APOE loci were identified as crucial factors for influencing the relationship between circulating metabolites and immune cell phenotypes through the bioinformatic analyses. Furthermore, CD33 and APOE were indicated as key genes involved in establishing the association between circulating metabolites and the risks of AD in an immune cell phenotype-dependent manner. The gene mutation for APOE has the ability to affect AD [42]. These accumulating pieces of evidence indicated that genes with the characteristics of influencing serum total cholesterol and immune cell traits can affect AD.

Based on the aforementioned “cholesterol-CD33-SHP-1/JAK1/STAT6” pathological axis, this study further explored the intervention value of TP, with its mechanism focusing on “liver-specific regulation of cholesterol synthesis”, a design closely aligned with the tissue-specific characteristics of cholesterol metabolism. It was observed that TP significantly inhibited the protein expression of SREBP2 and HMGCR in the liver of PS19 mice, but not in the brain. As the primary organ for peripheral cholesterol synthesis, the liver relies on the SREBP2/HMGCR pathway as the core regulatory node for cholesterol biosynthesis [23].

In this study, we found that CD33 expression positively correlated with SHP-1 expression, and that CD33 knockdown reduced SHP-1 levels, placing CD33 upstream of SHP-1 in this regulatory axis. This is consistent with a previous study showing that CD33 can associate with SHP-1 in human cells [24]; however, whether murine CD33 directly binds SHP-1 remains to be determined. Nonetheless, our data demonstrate that CD33 regulates SHP-1 expression, which in turn modulates JAK1/STAT6 signaling and microglial polarization. In a study, it was confirmed that the CD33/SHP-1 signaling pathway is positively correlated with AD [43]. Unlike human CD33, murine CD33 lacks canonical ITIM motifs required for direct SHP-1 activation but contains a functional ITIM-like motif that mediates the myeloid cell signaling pathway, according to NCBI. In our study, knocking down CD33 can reduce the expression of SHP-1; that means CD33 not only activates SHP-1 but also increases the level of SHP-1. Notably, the effects of CD33 depend on triggering receptor expressed on myeloid cells 2 (TREM2) [44], which was confirmed to increase the phosphorylation of STAT6 and the level of Arg1 [45]. In our study, cholesterol-induced neuroinflammation models and CD33 knockout experiments demonstrate that CD33 modulates SHP-1 expression, which impairs JAK1/STAT6 activation and subsequent Arg1 transcription, impacting microglial polarization and neuroinflammation. Although the mechanisms of CD33 inducing neuroinflammation via regulating SHP-1 expression are determined by this study, the specific model pathway still needs further investigation. The specific inhibition of this pathway by TP efficiently reduced circulating cholesterol levels, which in turn downregulated the expression of CD33/SHP-1 in the brain, leading to the restoration of the signaling activity of JAK1/STAT6 and promoting M2 polarization of microglia.

In addition to modulating the CD33/SHP-1/JAK1/STAT6 axis via cholesterol reduction, TP was also found to inhibit the CCAAT/enhancer-binding protein β (C/EBPβ)/asparagine endopeptidase (AEP) signaling pathway [22]. The C/EBPβ/AEP pathway serves as a key node linking metabolic disorders to neuroinflammation in AD; on the other hand, its activation can promote the release of pro-inflammatory cytokines and exacerbate tau pathology [46]. In this study, TP treatment significantly reduced the expression of CD33 in the brain of PS19 mice, decreasing the release of pro-inflammatory cytokines such as IL-1β. These studies support that TP can reduce neuroinflammation through regulating metabolism, which provides a novel insight into the anti-inflammation effects of TP.

Despite our findings demonstrating that TP alleviates neuroinflammation in AD by reducing cholesterol synthesis via a CD33-dependent mechanism, further investigation is required to address the remaining unresolved issues in the future. First, the present study focused on the regulatory role of total cholesterol in the CD33-mediated neuroinflammatory axis, while the specific effects of different cholesterol subfractions, such as LDL and HDL, etc., warrant further investigation. Second, the direct physical interaction between mouse CD33 and SHP-1 has not yet been confirmed by co-immunoprecipitation, leaving room for further verification at the biochemical level. Third, the impact of TP on peripheral immune cells was not fully characterized, and the long-term safety as well as the pharmacokinetic profile of intranasal TP administration for AD treatment require more detailed evaluation in future studies.

## 5. Conclusions

Collectively, TP alleviates cognitive impairment and neuroinflammation in PS19 tauopathy mice by inhibiting the hepatic SREBP2/HMGCR pathway, thereby reducing cholesterol levels. Lowering the levels of cholesterol suppress the CD33/SHP-1 axis, restore JAK1/STAT6 signaling, and shift microglial polarization from M1 to M2 phenotype. Our findings shed lights on the mechanism by which TP treats AD through the CD33/SHP-1/JAK1/STAT6 pathway in a cholesterol-dependent manner.

## Data Availability

All experimental data supporting the findings of this study are declared available within the article by authors. The primary data, materials, detailed information and codes required to reanalyze the data in this work are available from the corresponding authors upon reasonable request.

## Supplementary Materials

The following supporting information can be downloaded at: https://www.mdpi.com/article/10.3390/biology15110818/s1.

## Abbreviations

AAV	adeno-associated virus
AD	Alzheimer’s disease
AEP	asparagine endopeptidase
AMPK	AMP-activated protein kinase
ANOVA	analysis of variance
APOE	apolipoprotein E
APP	amyloid precursor protein
Arg1	arginase-1
Aβ	β-amyloid protein
BBB	blood–brain barrier
CCL2	C-C motif chemokine ligand 2
CD33	sialic acid-binding Ig-like lectin 3
C/EBPβ	CCAAT/enhancer-binding protein β
CHOD-PAP	cholesterol oxidase-peroxidase aminophenazone
CNS	central nervous system
HCD	high-cholesterol diet
HDL	high-density lipoprotein
HMGCR	3-hydroxy-3-methylglutaryl-coenzyme A reductase
Iba1	allograft inflammatory factor-1
IHC	immunohistochemistry
IL-1β	interleukin-1β
ITIM	immunoreceptor tyrosine-based inhibitory motif
JAK1	Janus kinase 1
LDL	low-density lipoprotein
NF-κB	nuclear factor kappa-B
NOR	novel object recognition
Nrf2	nuclear factor erythroid 2-related factor 2
PBS	phosphate-buffered saline
PVDF	polyvinylidene fluoride
SDS-PAGE	sodium dodecyl sulfate polyacrylamide gel electrophoresis
S.E.M.	standard error of the mean
SHP-1	SH2-containing protein tyrosine phosphatase-1
SREBP2	sterol regulatory element-binding protein 2
STAT6	signal transducer and activator of transcription 6
TP	triptolide
TREM2	triggering receptor expressed on myeloid cells 2
VLDL	very low-density lipoprotein
WB	western blot
WT	wild-type
