# Supplementary material for: Triptolide Reduces Cholesterol Synthesis and Alleviates Neuroinflammation by Inhibiting CD33 in Alzheimer’s Disease Development and Progression
Source: Biology (Basel). 2026 May 22;15(11):818. doi: 10.3390/biology15110818 (PMC13255628; doi:10.3390/biology15110818)
Supplement: Supplementary file 1 [file biology-15-00818-s001.zip › ARRIVE_20260430.pdf]

# ARRIVE 2.0 Essential 10 and Recommended Set


## Abstract

The animal experiments in this study aimed to investigate the effects of triptolide (TP) in alleviating neuroinflammation via the cholesterol–CD33–SHP-1/JAK1/STAT6 axis. PS19 transgenic mice (a tauopathy model of AD), C57BL/6 wild-type (WT) mice, and AAV-mediated CD33 knockdown mice were used. WT mice were fed either a standard diet or a high-cholesterol diet (HCD) to elucidate the role of cholesterol in AD-associated neuroinflammation. TP was intraperitoneal injection (I.P.) administered (20 μg/kg) to the PS19 mice. Primary outcome measures included behavioral assessments (Morris water maze, novel object recognition) and molecular pathologic analyses.


## Essential 10

**1. Study design**

**TP treatment experiments**: WT, PS19, and PS19 treated with TP.

**HCD and CD33 knockdown experiments**: WT, HCD, and HCD + AAV-shRNA-CD33 knockdown.

**2. Sample size**

**TP treatment experiment**: n = 5 male mice per group.

**HCD and CD33 knockdown experiment**: n = 4 male mice per group.

**3. Inclusion and exclusion criteria**

**Inclusion criteria:** PS19 mice positive mice and the littermate negative mice.

**Exclusion criteria:** (1) Death or obvious health problems (e.g., severe fight wounds, body weight loss > 20%, considered a humane endpoint) before the intervention began; the data from such animals would be excluded from analysis. (2) Inability to locate the visible platform for three consecutive days during Morris water maze training (exclusion of visual or motor dysfunction). (3) Macroscopic intracranial hemorrhage or other unexpected lesions found during sample collection.

**4. Randomisation**

Mice were randomly assigned to experimental groups using a computer-generated random number sequence with cage No. as a blocking factor. The randomisation procedure was performed by a colleague not involved in the subsequent experiments, to ensure allocation concealment.

**5. Blinding**

Behavioral testing (Morris water maze, novel object recognition) was performed by an experimenter blinded to group allocation. Image acquisition and analysis of tissue sections (Iba1 immunohistochemistry) and densitometric quantification of Western blots were also conducted under blinded conditions.

**6. Outcome measures**

Primary outcome measures: The number of escape latency during water maze training, platform crossings in the Morris water maze probe trial; the recognition index in the novel object recognition test.

Secondary outcome measures: Iba1-positive microglial morphological activation (qualitative, IHC); protein expression levels of CD33, APOE, IL-1β, Arg1, SHP-1, p-JAK1/JAK1, and p-STAT6/STAT6 in brain tissues (Western blot).

**7. Statistical methods**

Data presentation: All data are expressed as means ± S.E.M.

Statistical tests: Comparisons between two groups were performed using an unpaired two-tailed Student's t-test. Comparisons among multiple groups were performed using one-way analysis of variance (ANOVA) followed by Tukey's post hoc multiple comparisons test.
Significance level: A $p$-value $< 0.05$ was considered statistically significant. Exact $p$-values are indicated by asterisks in the figures as explained in the figure legends.
Software: All analyses were performed using GraphPad Prism version 10.4.1

**8. Experimental animals**

**Species/strain:** PS19 (B6;C3-Tg (Prnp-MAPT*P301S)PS19Vle/J; Stock No. 024841) and wild-type C57BL/6J mice were obtained from Cavens (Changzhou, Jiangsu, China).
**Sex, age, and weight**: All male, 4 months old at the start of the experiment, weighing approximately 18-20 g before the beginning of treatment.
**Housing conditions**: Animals were housed in an SPF barrier facility, 3–4 per cage, at 22 ± 1°C and 50%–60% relative humidity, under a 12-hour/12-hour light/dark cycle. Mice had *ad libitum* access to food and water, standard bedding, and paper as nesting material.
**Acclimatisation**: All animals were allowed to acclimatise to the housing conditions for at least **7 days** before experimental interventions began.

**9. Experimental procedures**

**HCD**: Starting at 4 months of age, mice were fed a custom high-cholesterol diet containing 1.25% cholesterol (please verify the exact diet formula and product number) for 8 weeks.
**TP administration**: Starting at 5 months of age, PS19 mice received 20 µg/kg TP (dissolved in 1% DMSO, concentration and volume) I.P. every two days for 4 months. The control group received an equal volume of vehicle.
**CD33 knockdown**: Adeno-associated virus carrying AAV-shRNA-CD33 was used to knock down CD33; the control group received an AAV carrying a scrambled sequence.
**Behavioral tests**: Detailed parameters for the Morris water maze (day 1 visible platform, days 2–7 hidden platform training, day 8 probe trial) and novel object recognition test (5 min familiarisation, 1 h retention, 5 min test phase) have been described in the original sections 2.3 and 2.4.
**Anaesthesia and euthanasi**a: After behavioral testing, mice were deeply anesthetised with injection of urethane (2.0 g/kg body weight; 20% w/v solution in sterile saline, prepared fresh) and transcardially perfused with 0.9% saline, after which they were euthanized and the brain and liver were collected for subsequent analyses.
**humane endpoints**: Animals were observed daily. Pre-specified humane endpoints included: body weight loss exceeding 20%, persistent hunched posture, inability to eat or drink autonomously, or severe locomotor difficulties. Any animal reaching a humane endpoint was immediately removed from the experiment and euthanized as described above. Any adverse effects related to TP administration must be documented.

**10. Results**

For **TP treatment experiment**, the main results are presented in Figure 1: compared with the PS19 model group, the TP-treated group showed a significantly increased number of platform crossings in the Morris water maze probe trial, and a significantly higher recognition index in the novel object recognition test.
For **HCD and CD33 knockdown experiment**, the effects of CD33 knockdown are illustrated in Figure 2: compared with the HCD model group, the CD33 knockdown group showed improved cognitive function, along with altered expression of CD33 and its downstream pathway proteins in

the brain (see Figures 2G–H and 5C–D).
Exact *p*-values, effect sizes, and precise n numbers for all data are provided in the respective figure legends.

**Recommended Set**
**11. Abstract**
See the Abstract section.
**12. Background**
The scientific premise and necessity of the study have been elaborated in the Introduction.
**13. Objectives**
The study objectives to validate the "cholesterol–CD33–SHP-1/JAK1/STAT6" axis and evaluate the interventional effect of TP, are clearly stated.
**14. Ethical statement**
The "Ethics approval" section at the end of the manuscript states that the protocol was approved by the Laboratory Ethics Committee of Northeastern University (Approval No: **NEU-EC-2023A029S, Approval Date:22th May 2023**).
**15. Housing and husbandry**
Details have been supplemented in Essential 10, item 8.
**16. Animal care and monitoring**
Details have been supplemented in Essential 10, item 9.
**17. Scientific interpretation**
The results have been discussed in the context of the existing literature, and limitations of the methodology have been acknowledged in the Discussion.
**18. Generalisability/translation**
The Discussion mentions the interventional significance of CD33-regulated M2 polarization in neuroinflammation and highlights the translational potential of this axis as a therapeutic target.
**19. Protocol registration**
Not registered
**20. Data access**
"Availability of data and materials" states that the data are available from the corresponding author upon reasonable request.
**21. Declaration of interests**
**Declaration of interests:** No competing financial interests have been declared.

**Fund:** This work was supported by grants from Shenzhen Medical Research Fund (D2402007), Natural Science Foundation of Top Talent of SZTU (GDRC202404) and Special projects in key areas of ordinary universities in Guangdong Province (2025ZDZX2061).
