# Supplementary material for: Triptolide Reduces Cholesterol Synthesis and Alleviates Neuroinflammation by Inhibiting CD33 in Alzheimer’s Disease Development and Progression
Source: Biology (Basel). 2026 May 22;15(11):818. doi: 10.3390/biology15110818 (PMC13255628; doi:10.3390/biology15110818)
Supplement: Supplementary file 1 [file biology-15-00818-s001.zip › WB_Original Bands.pdf]

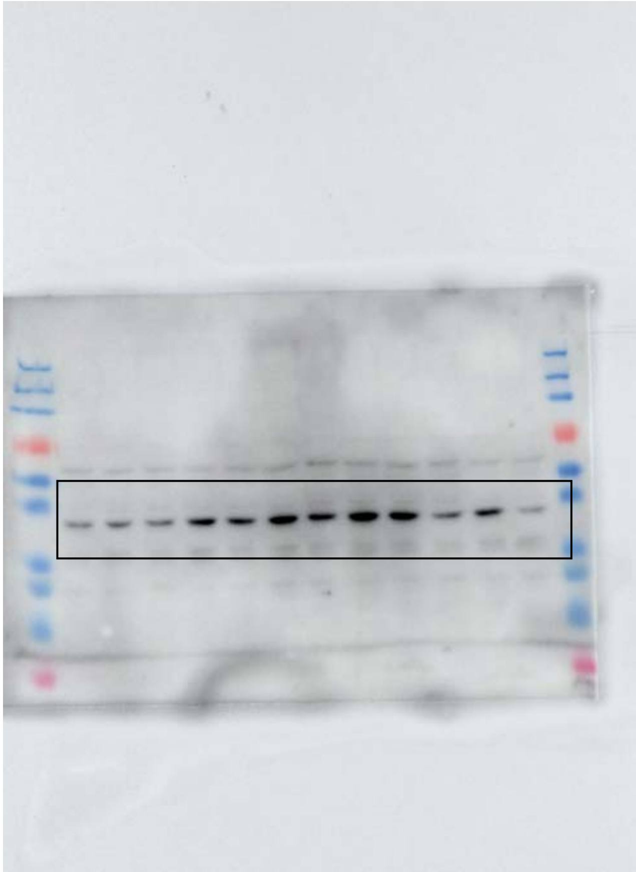

Figure 1F CD33

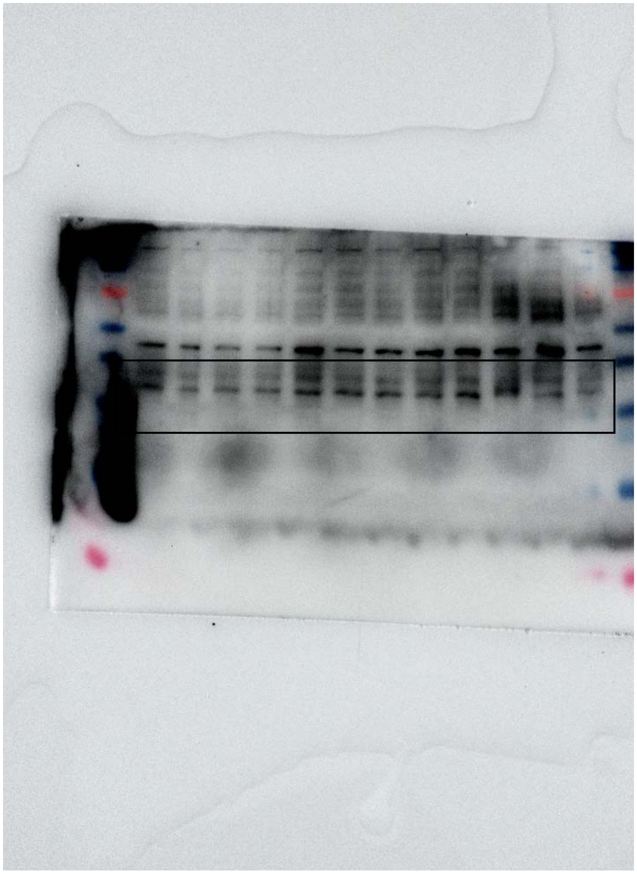

Figure 1F APOE

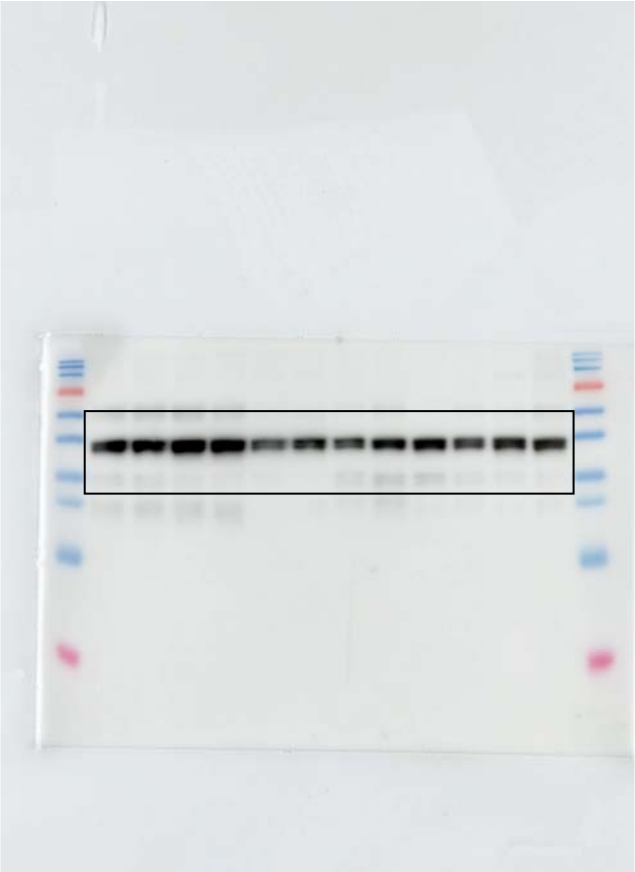
Figure 1F Arg1

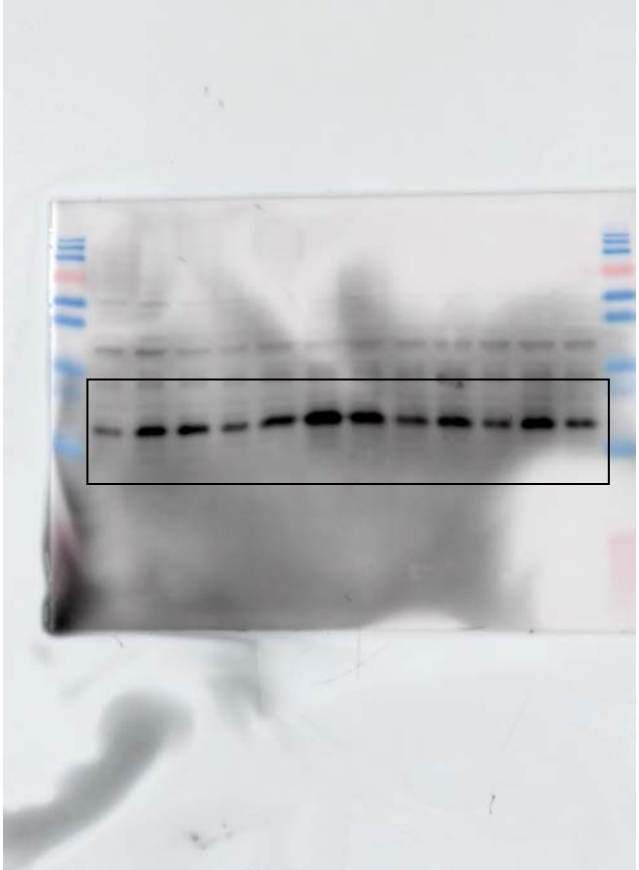
Figure 1F IL-1β

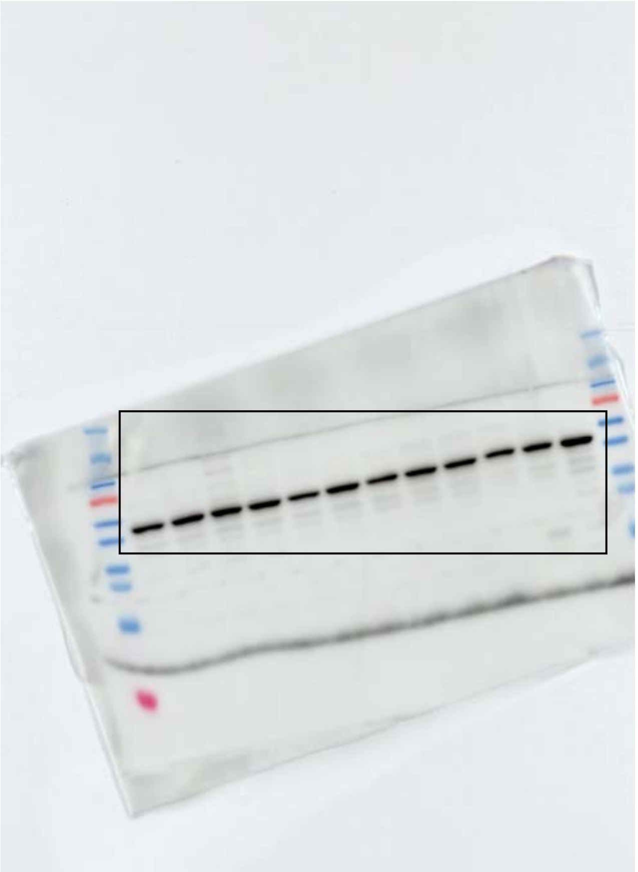

Figure 1F β-actin

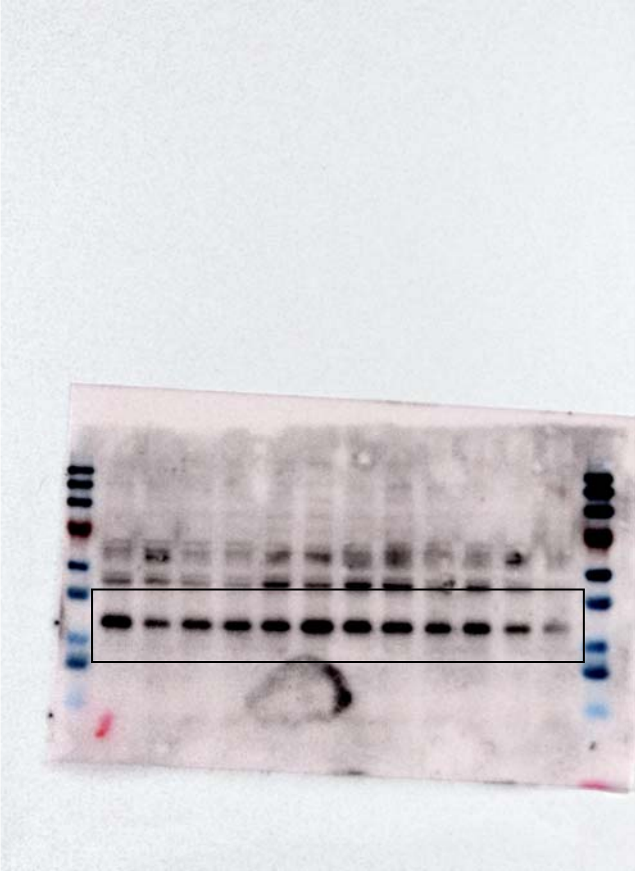

Figure 1G CD33

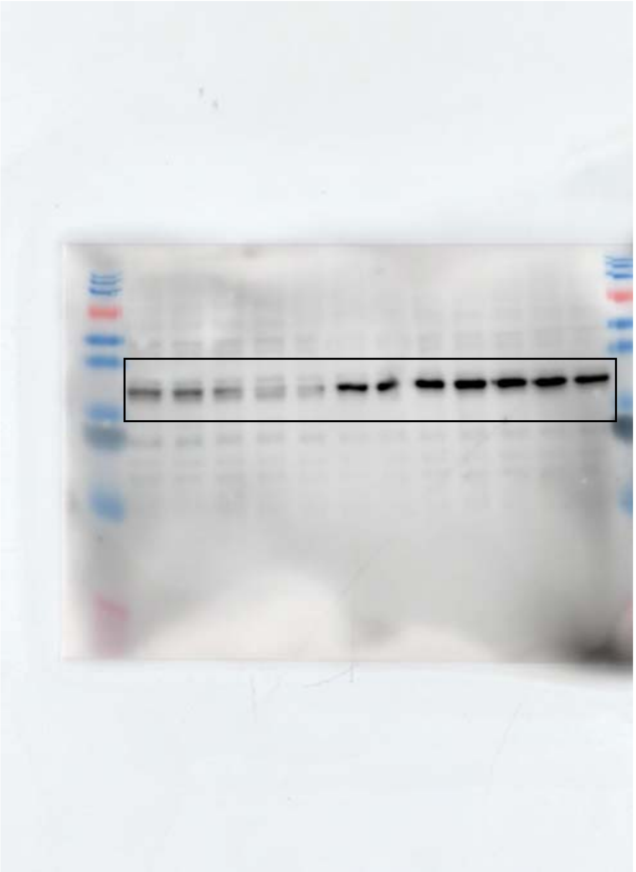
Figure 1G APOE

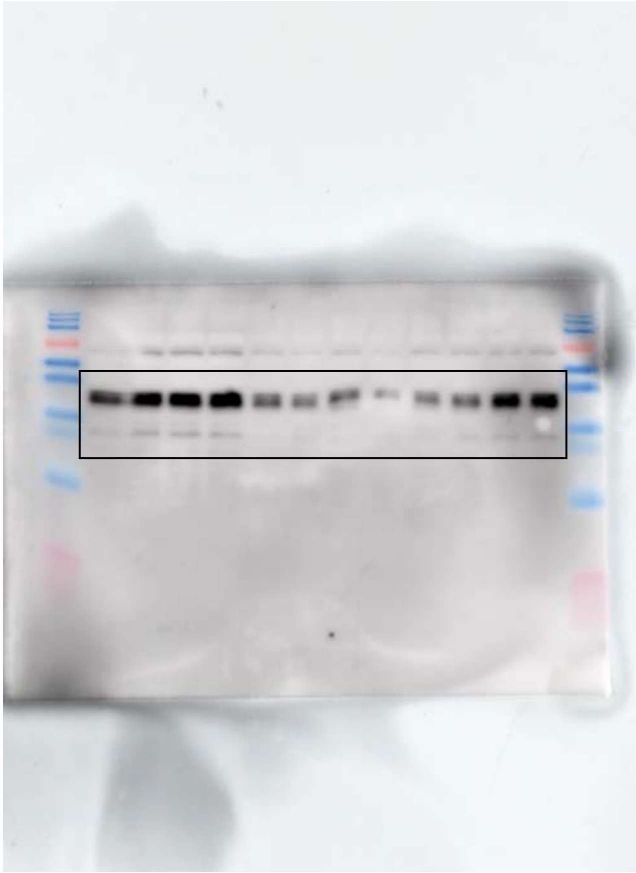
Figure 1G Arg1

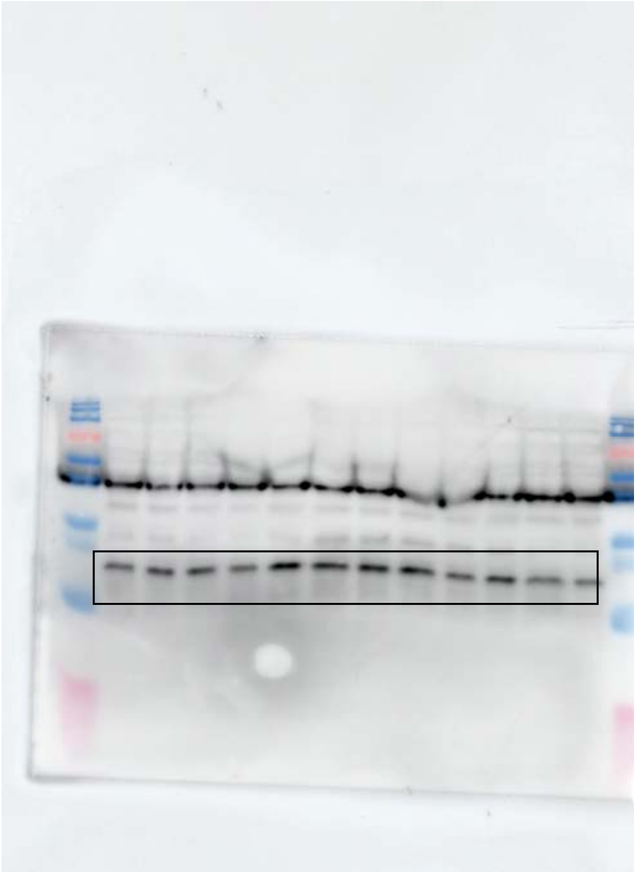

Figure 1G IL-1β

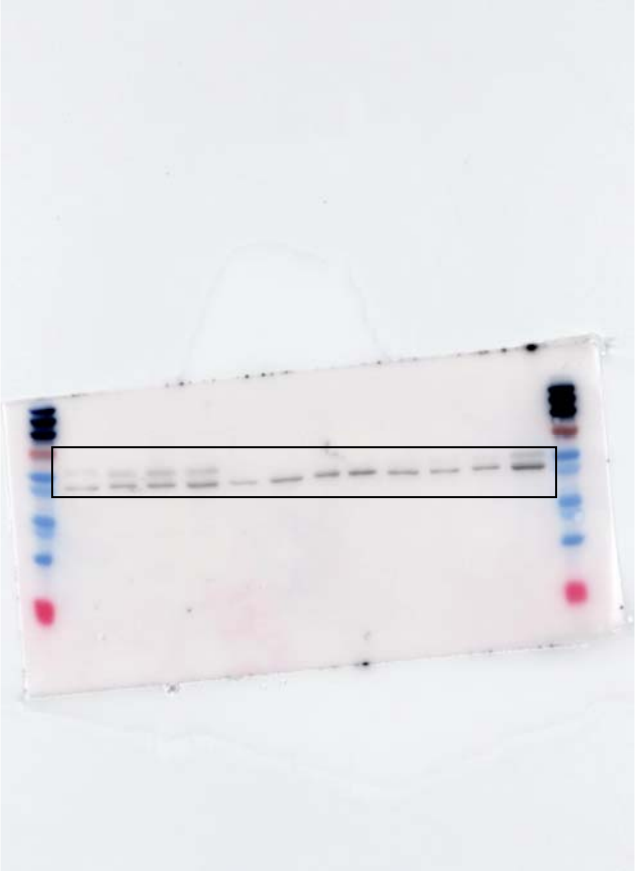

Figure 1G β-actin

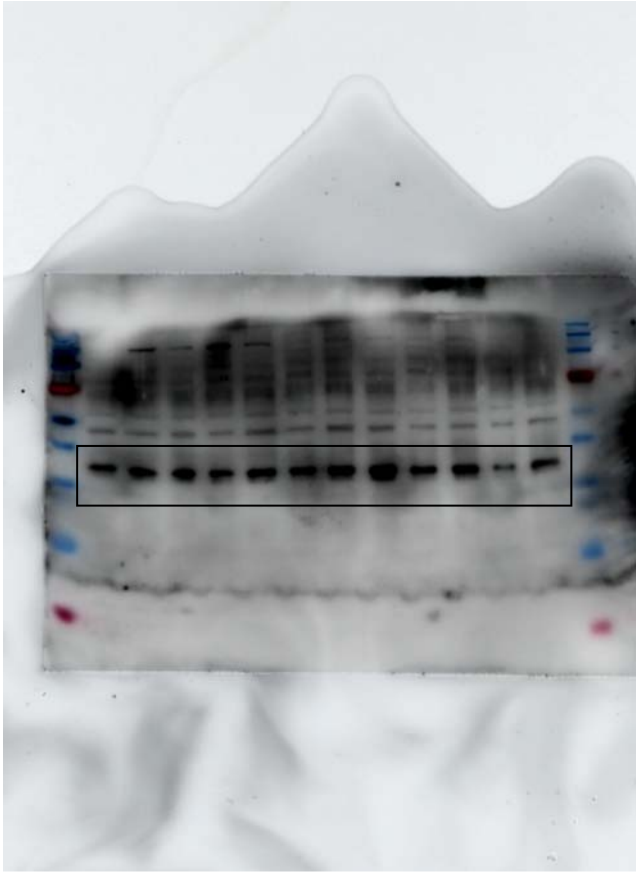

Figure 2G CD33

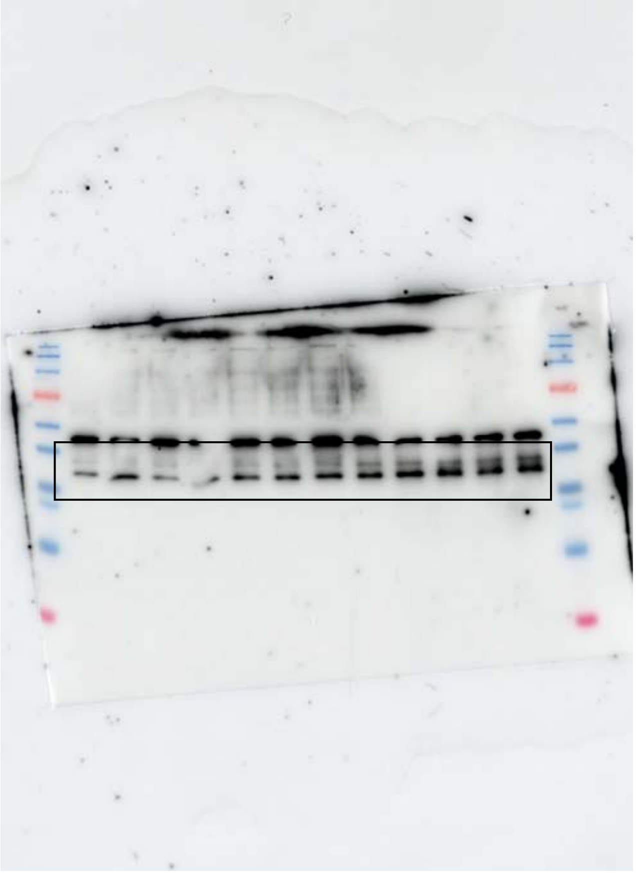

Figure 2G APOE

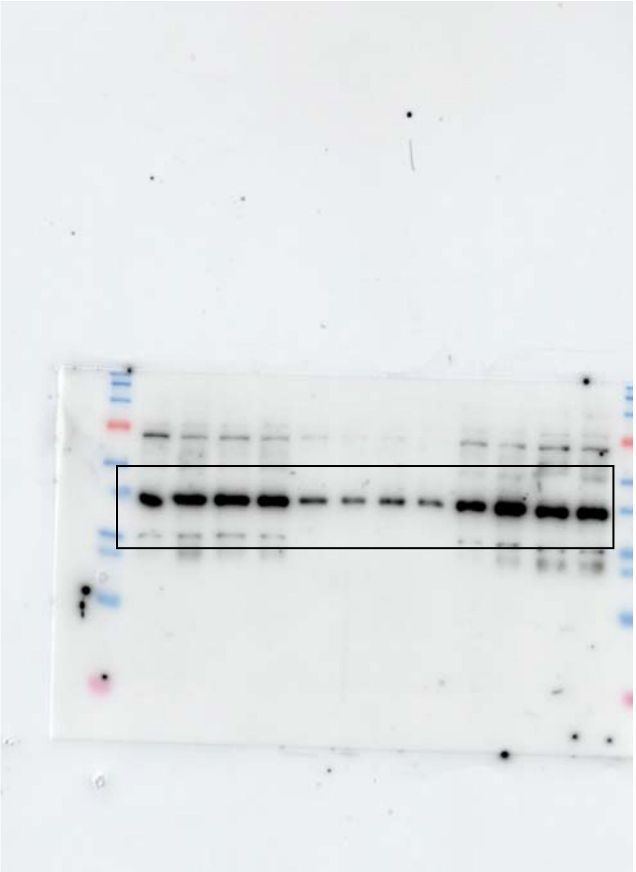

Figure 2G Arg1

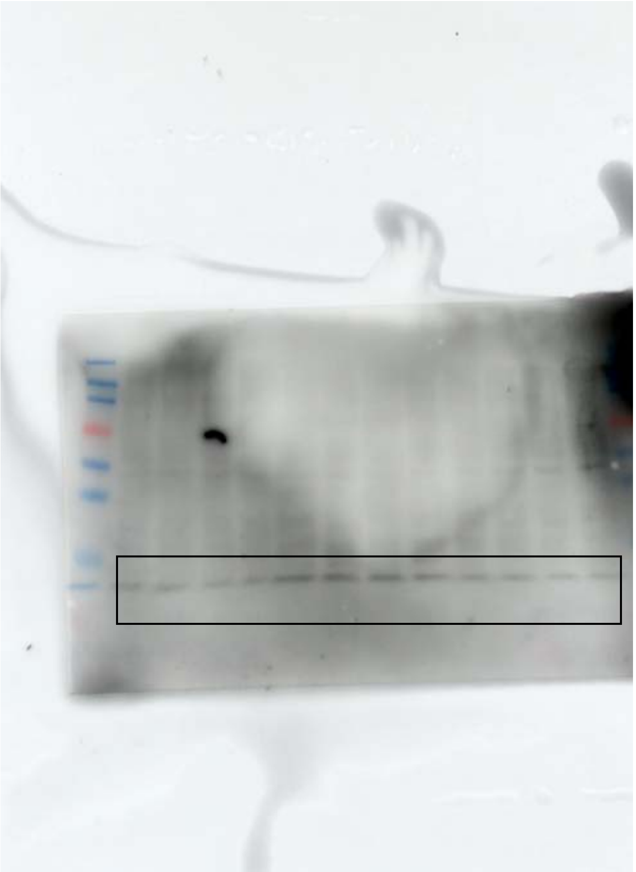

Figure 2G IL-1β

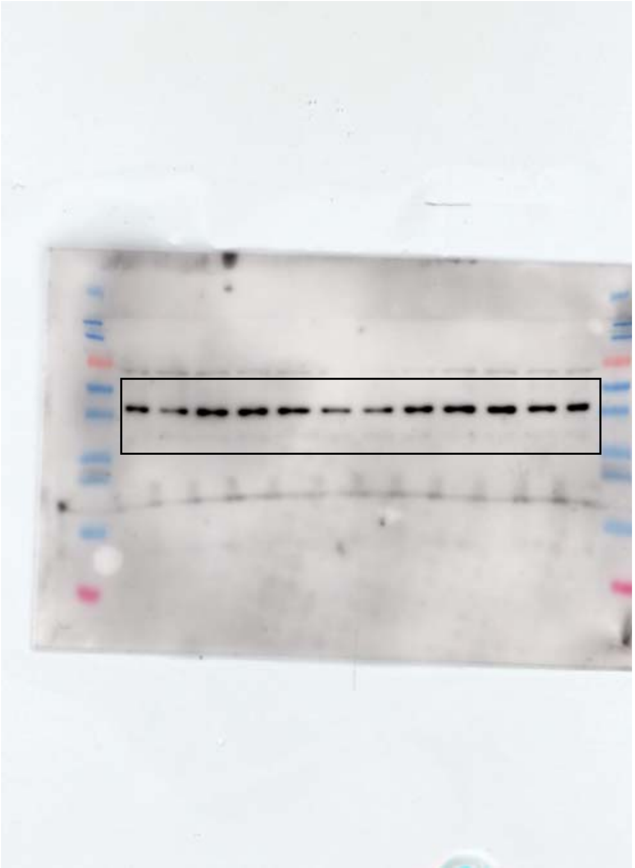

Figure 2G β-actin

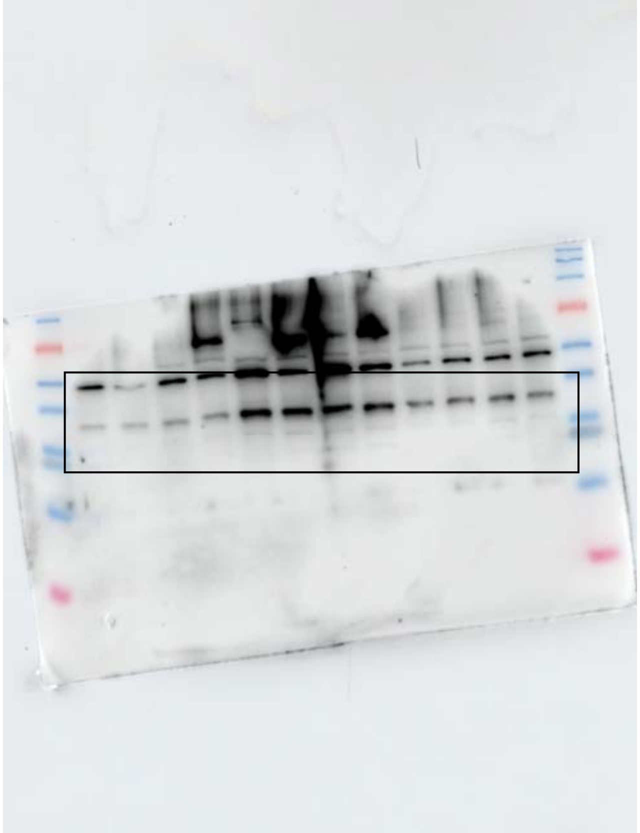

Figure 2H CD33

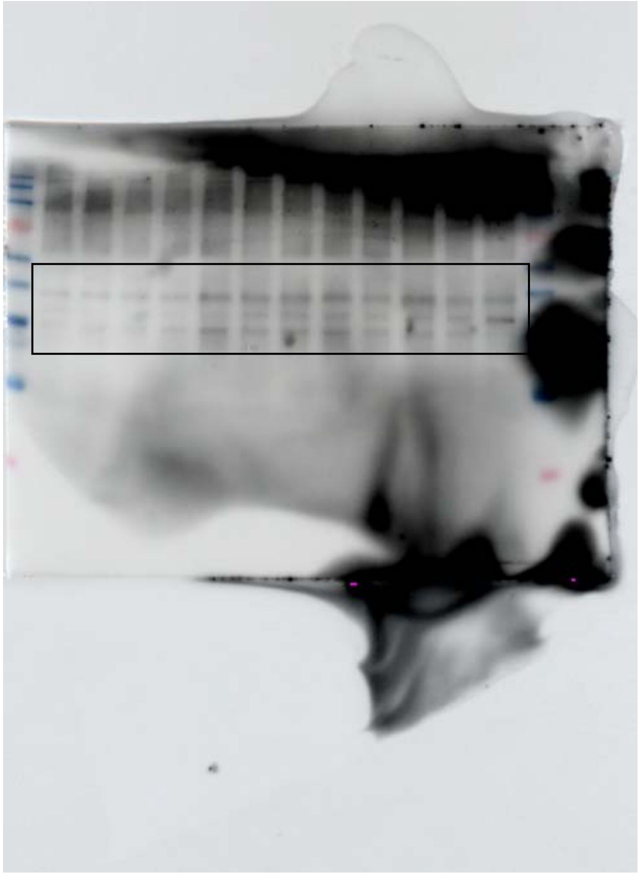

Figure 2H APOE

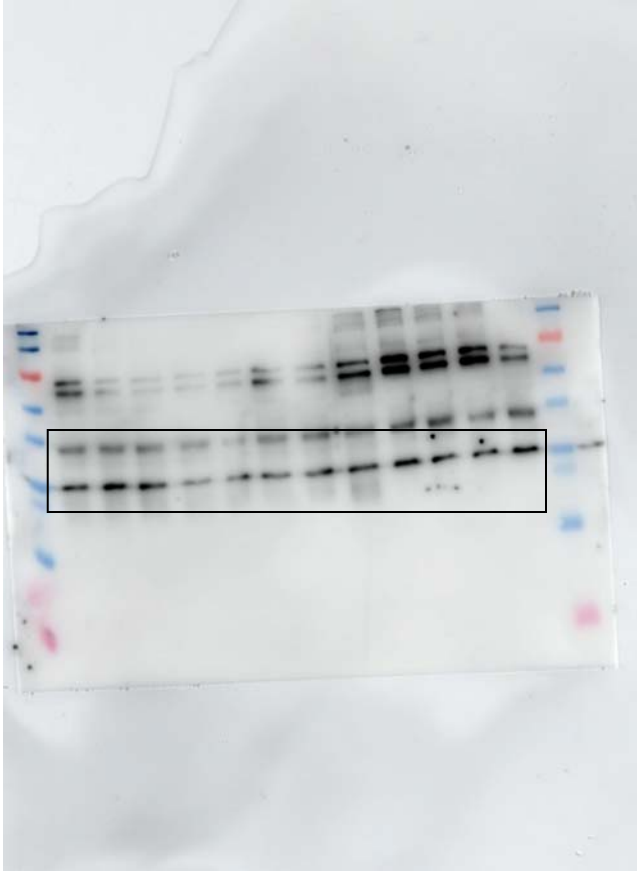

Figure 2H Arg1

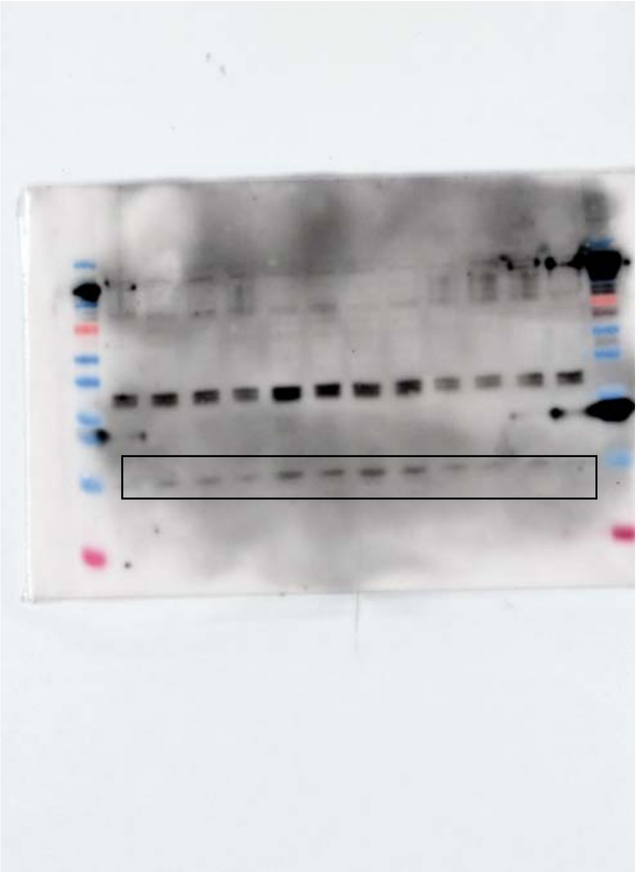

Figure 2H IL-1β

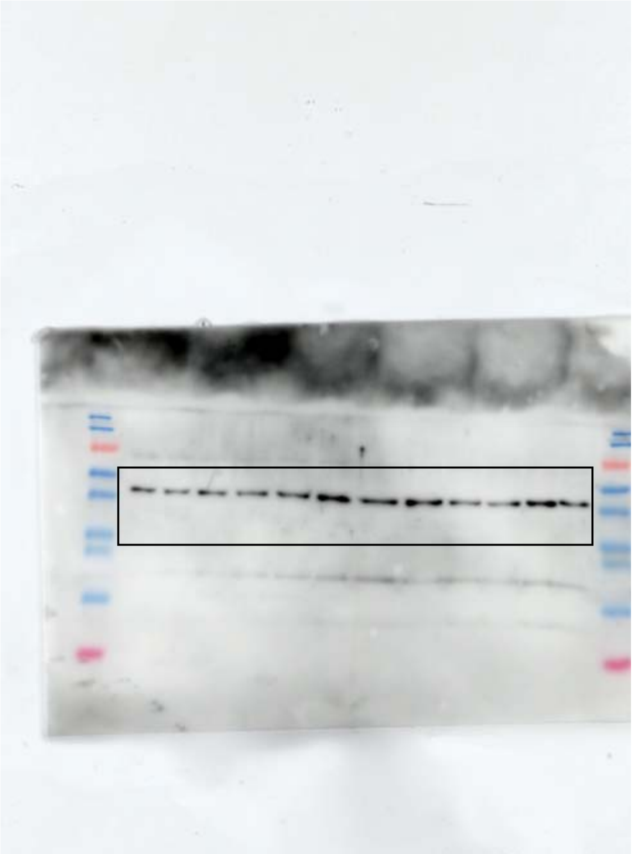

Figure 2H β-actin

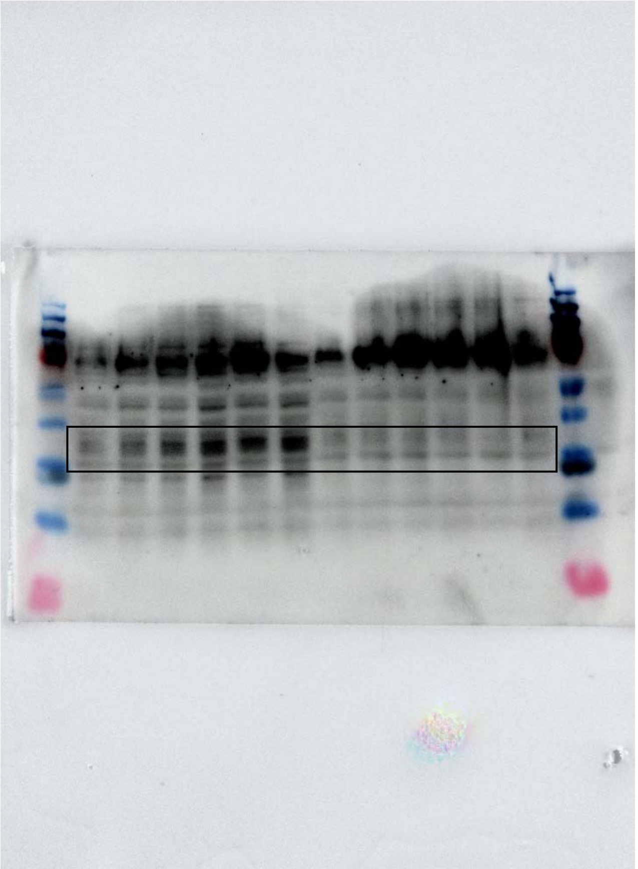

Figure 3A CD33

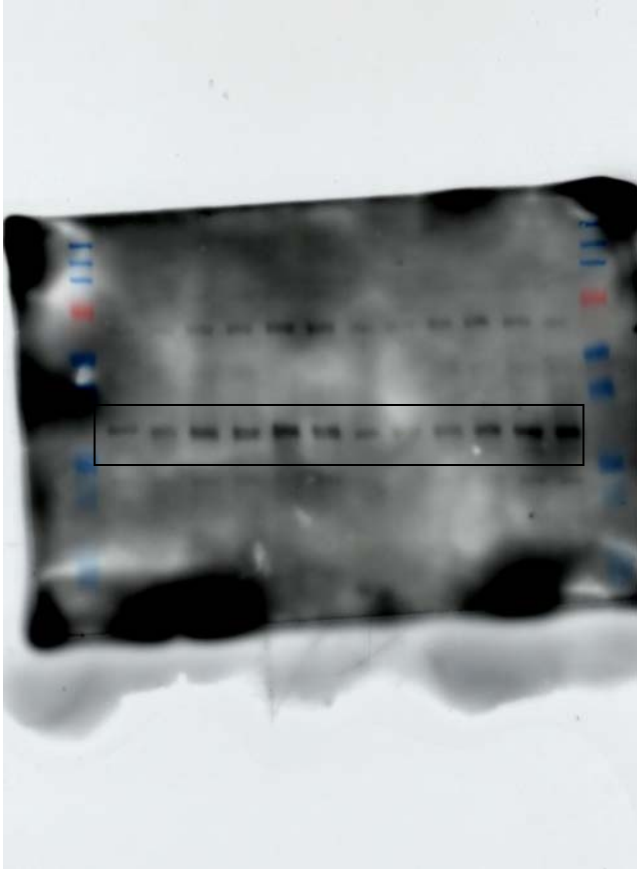

Figure 3A APOE

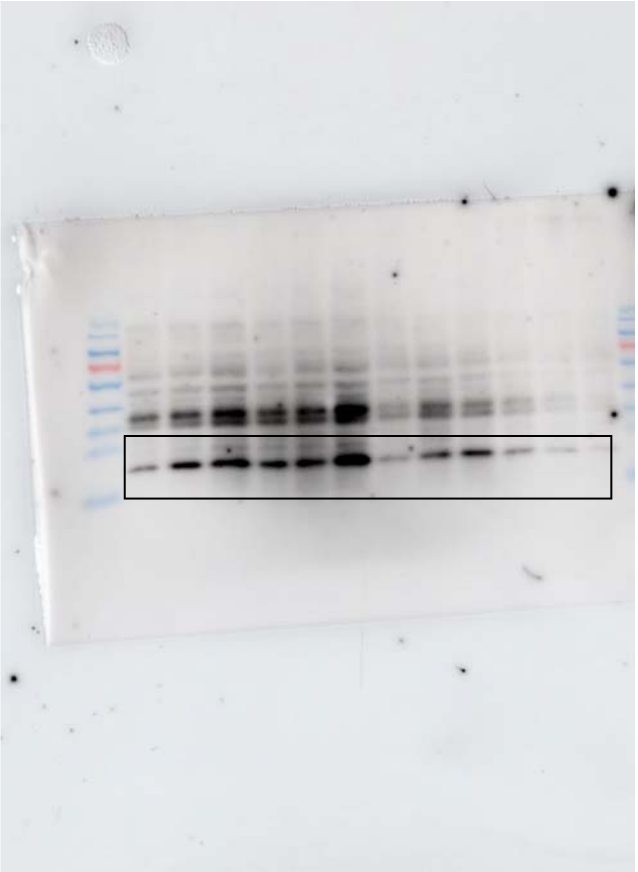

Figure 3A IL-1β

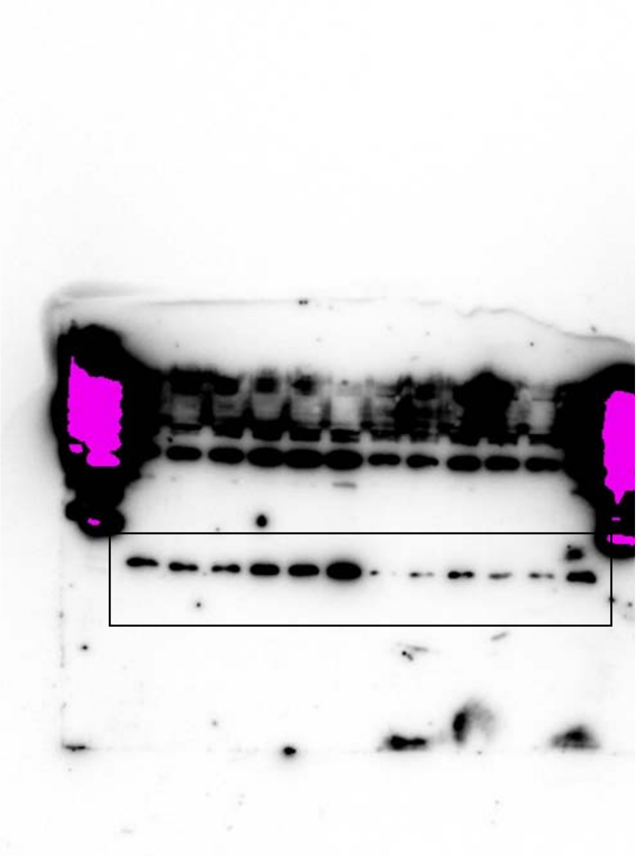

Figure 3A Iba1

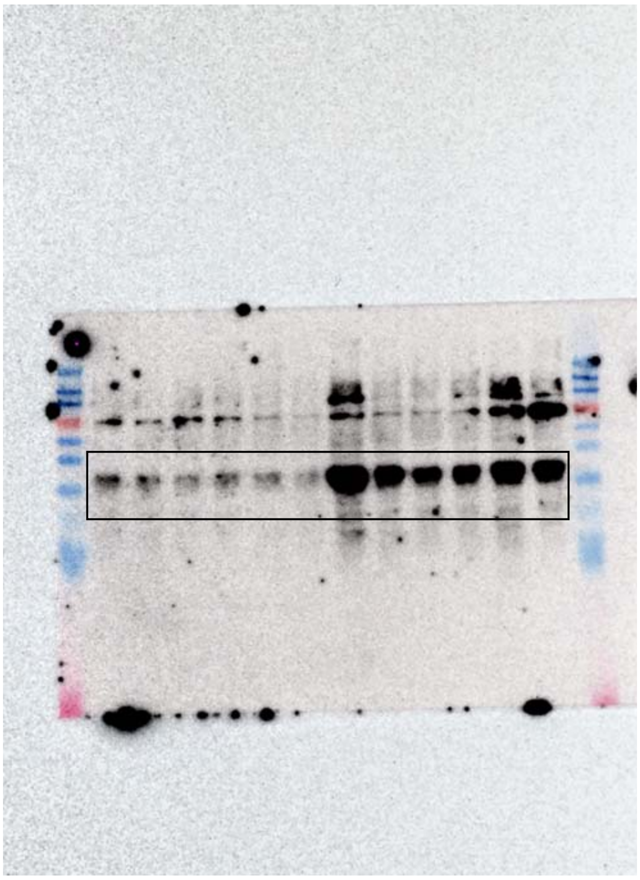

Figure 3A Arg1

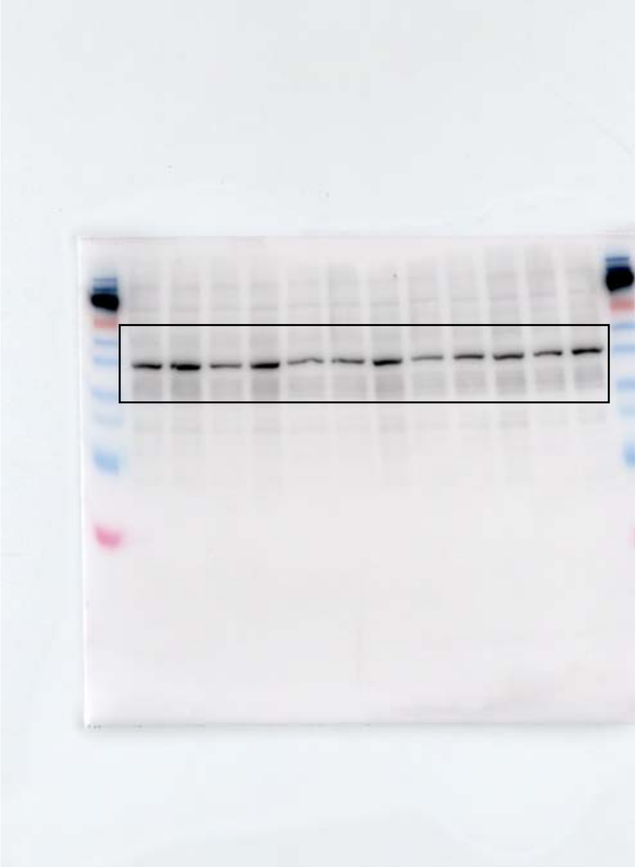

Figure 3A β-actin

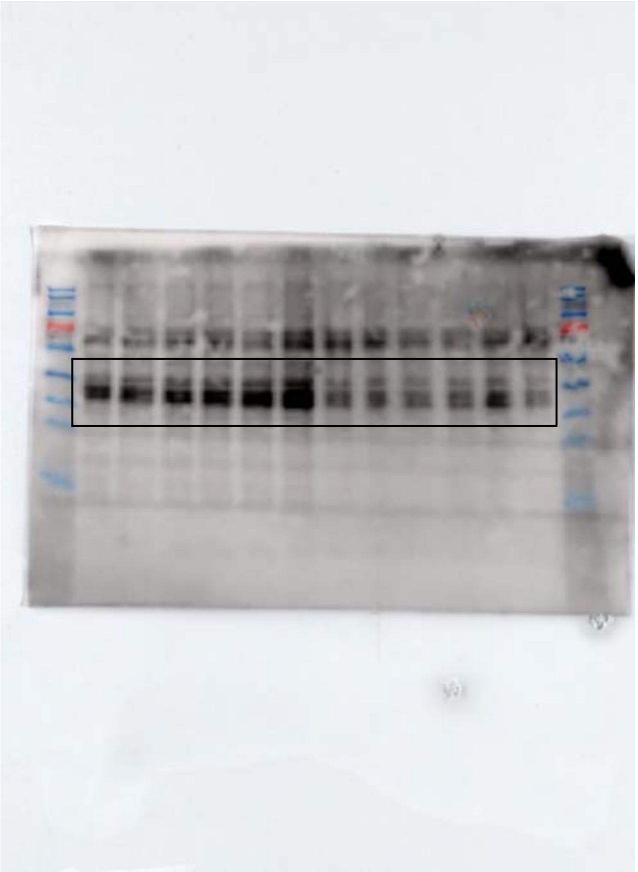

Figure 3B CD33

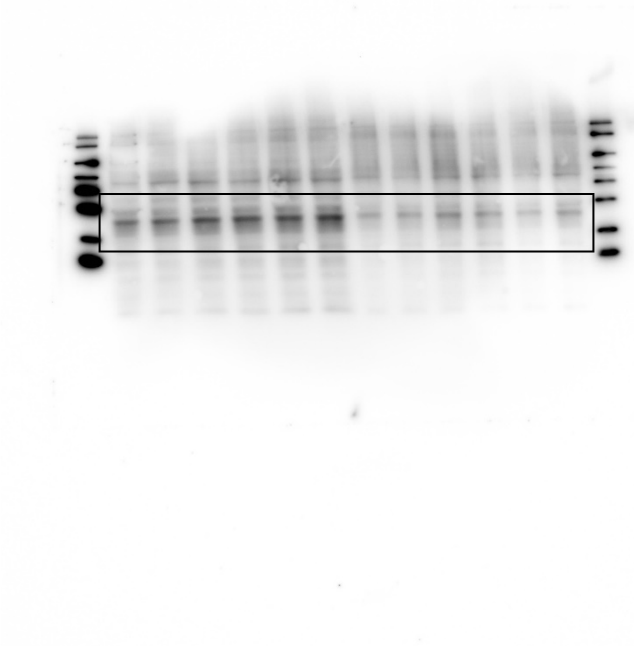

Figure 3B APOE

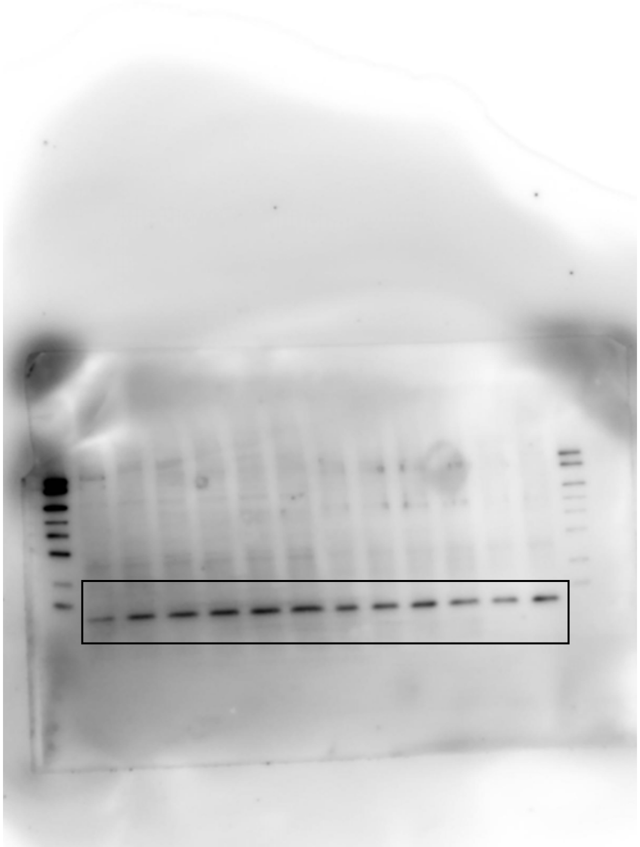

Figure 3B IL-1β

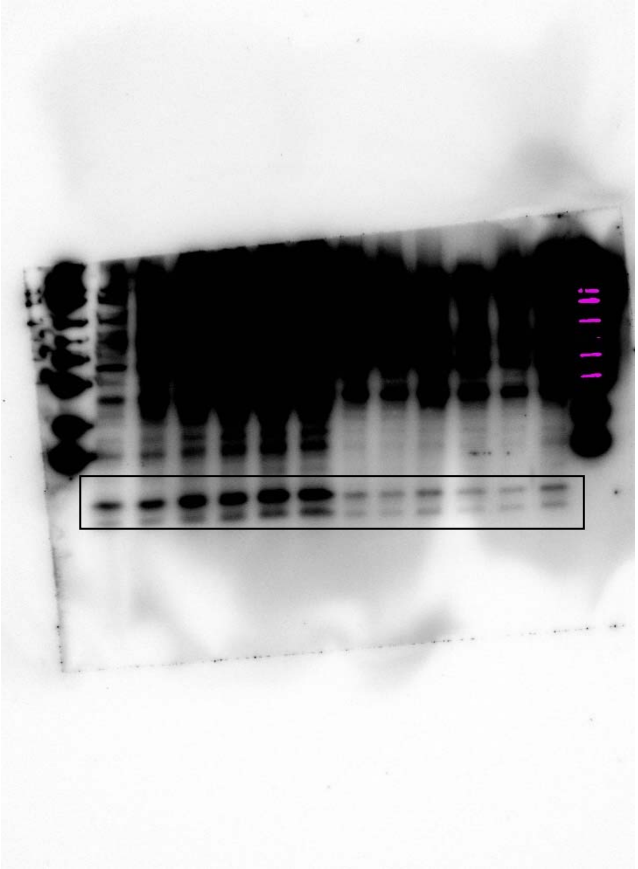

Figure 3B Iba1

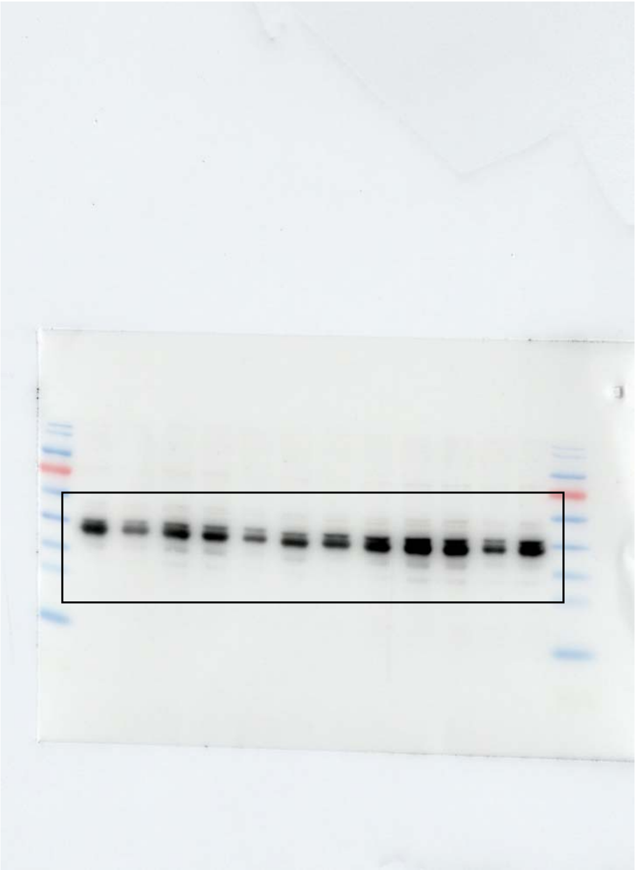

Figure 3B Arg1

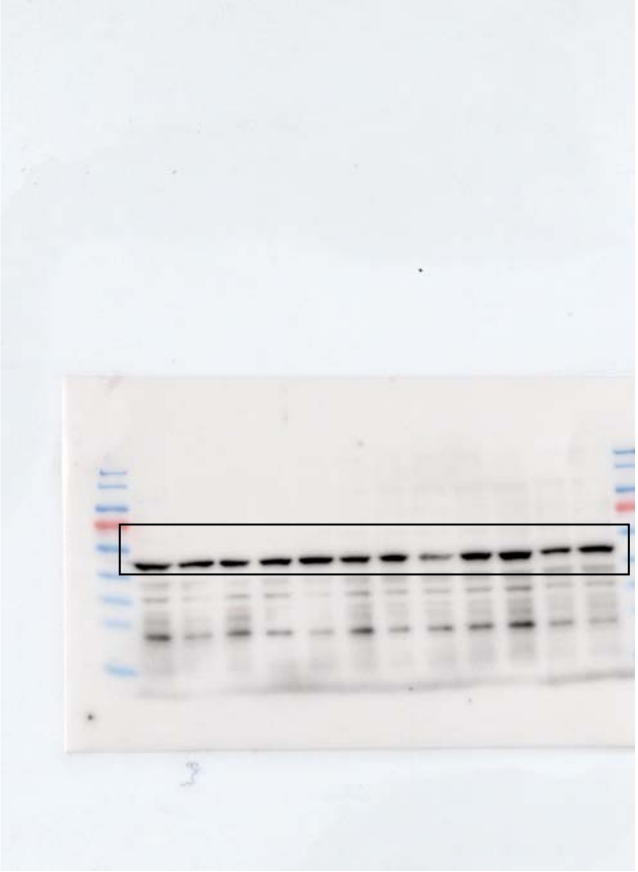

Figure 3B β-actin

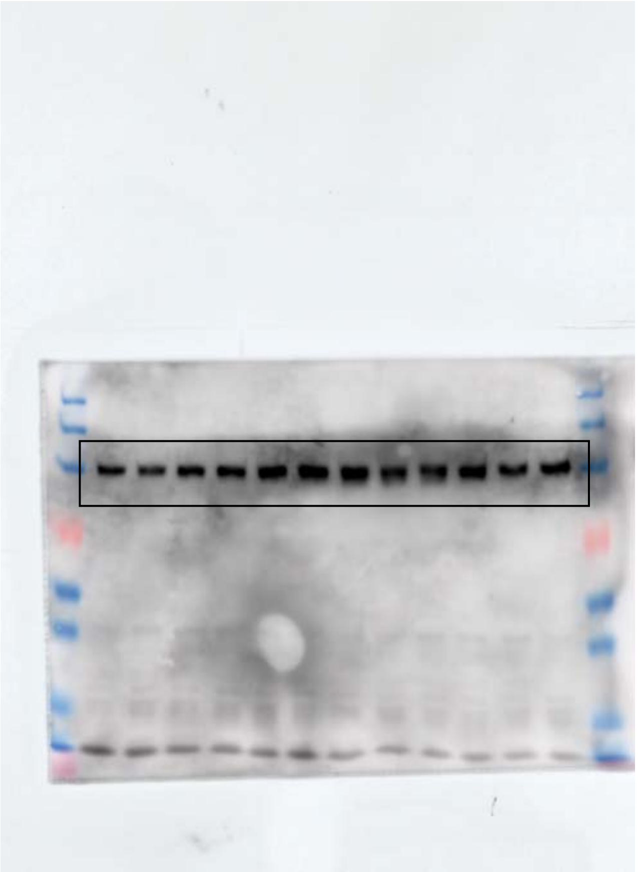

Figure 4A HMGCR

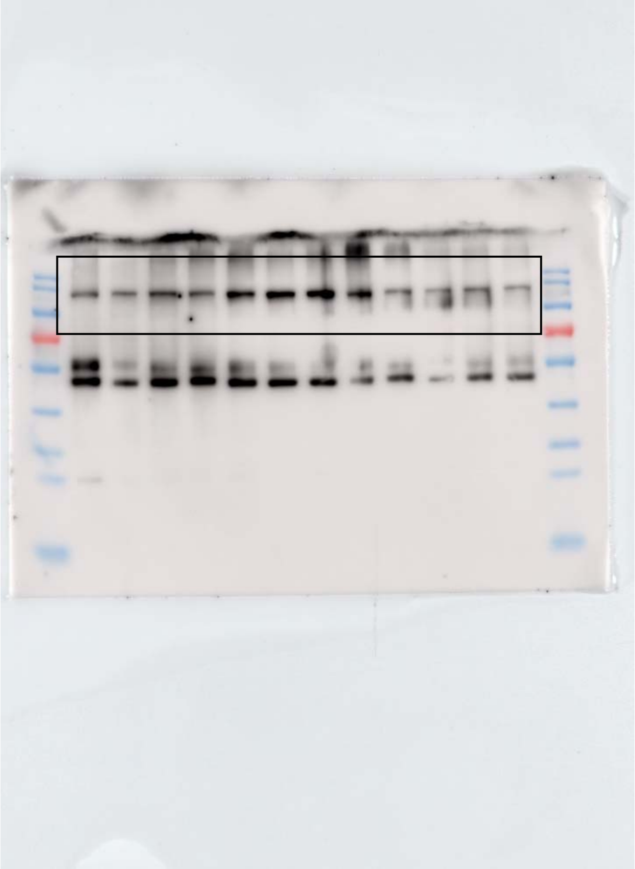

Figure 4A SREBP2

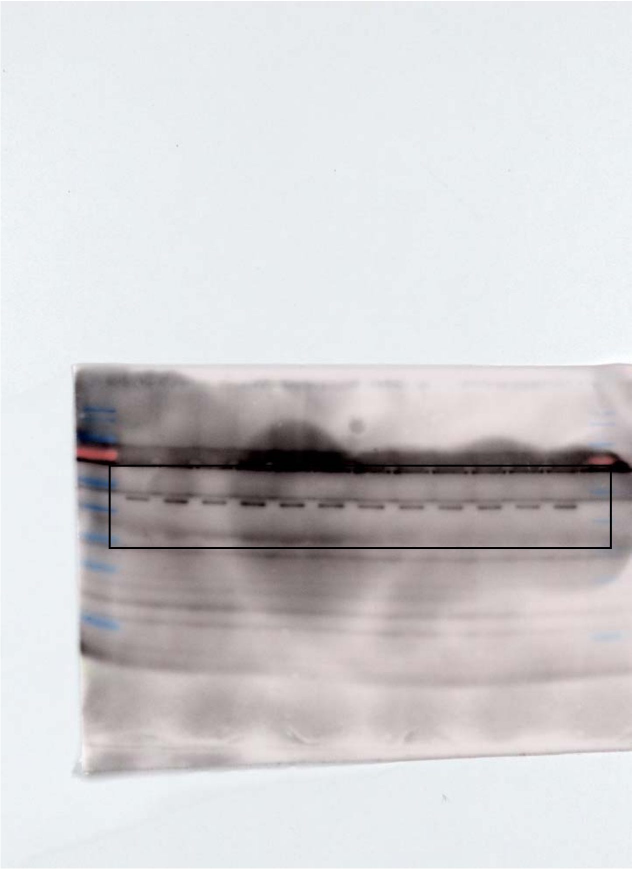

Figure 4A β-actin

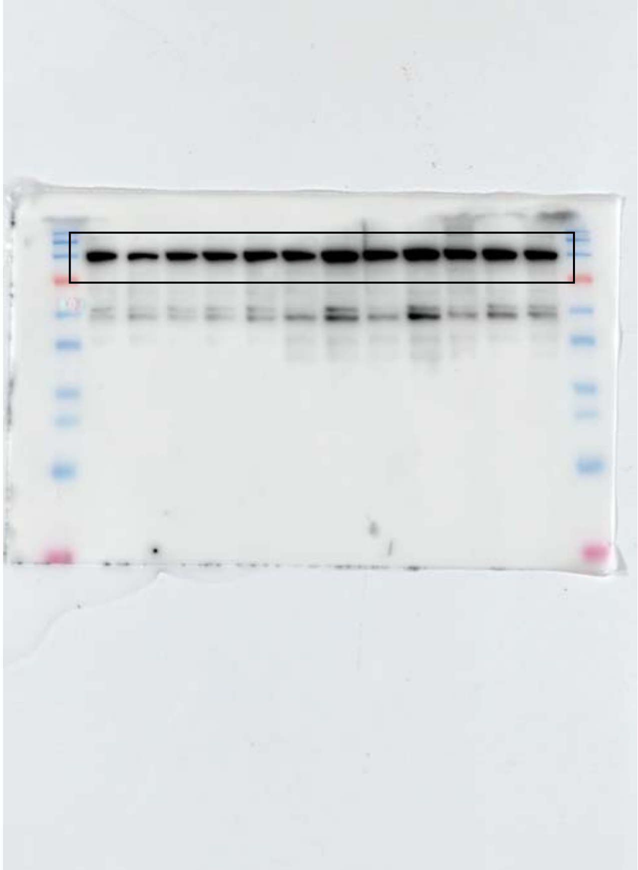

Figure 4B HMGCR

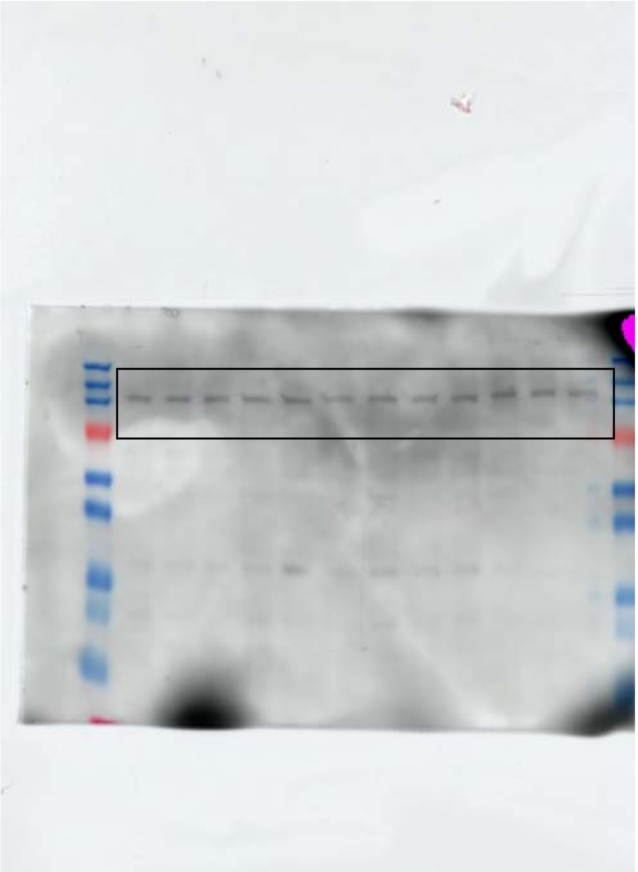

Figure 4B SREBP2

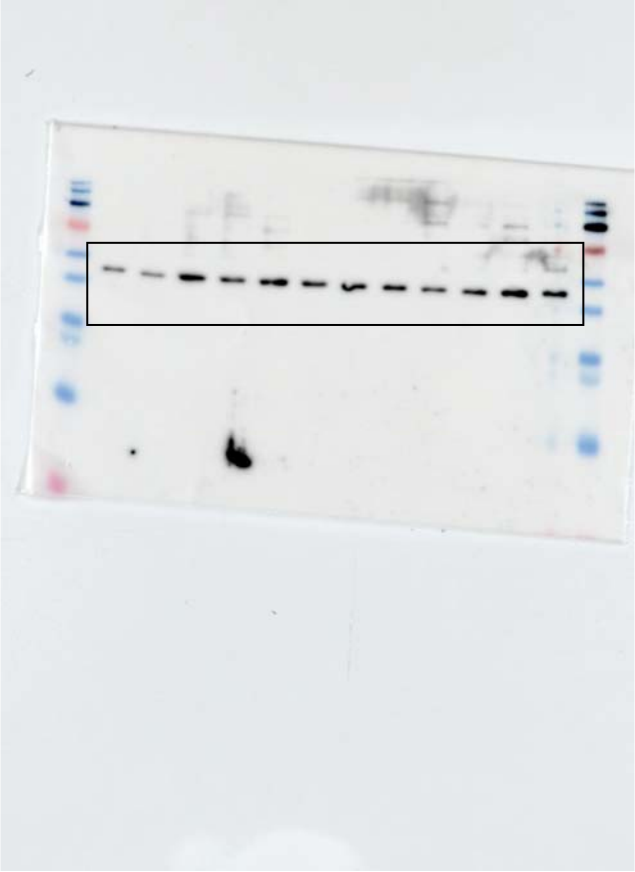

Figure 4B β-actin

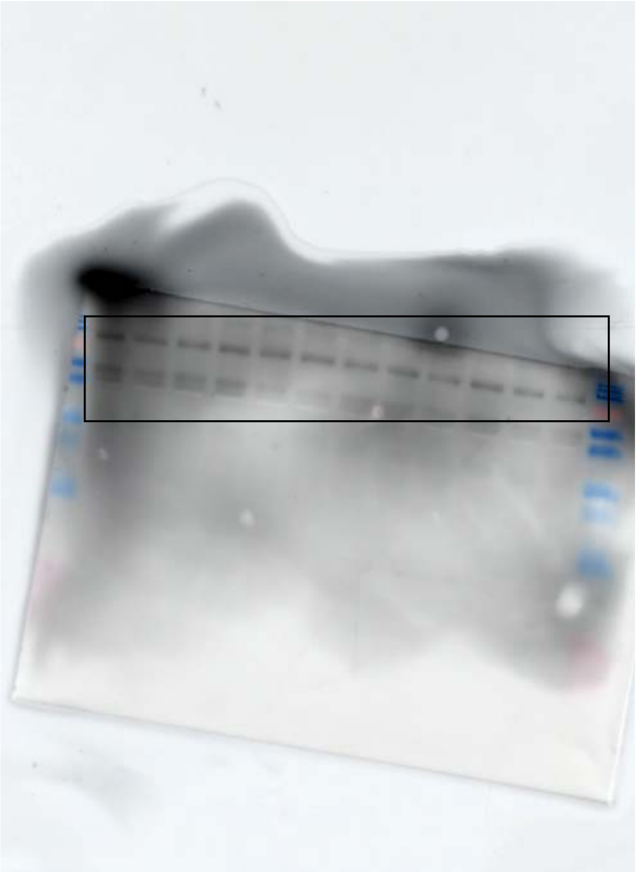

Figure 4C HMGCR

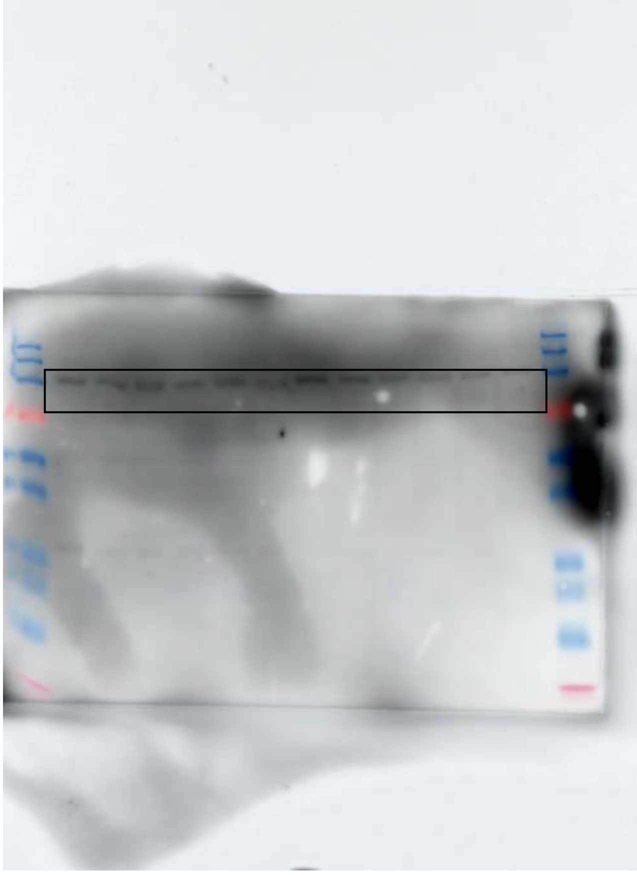

Figure 4C SREBP2

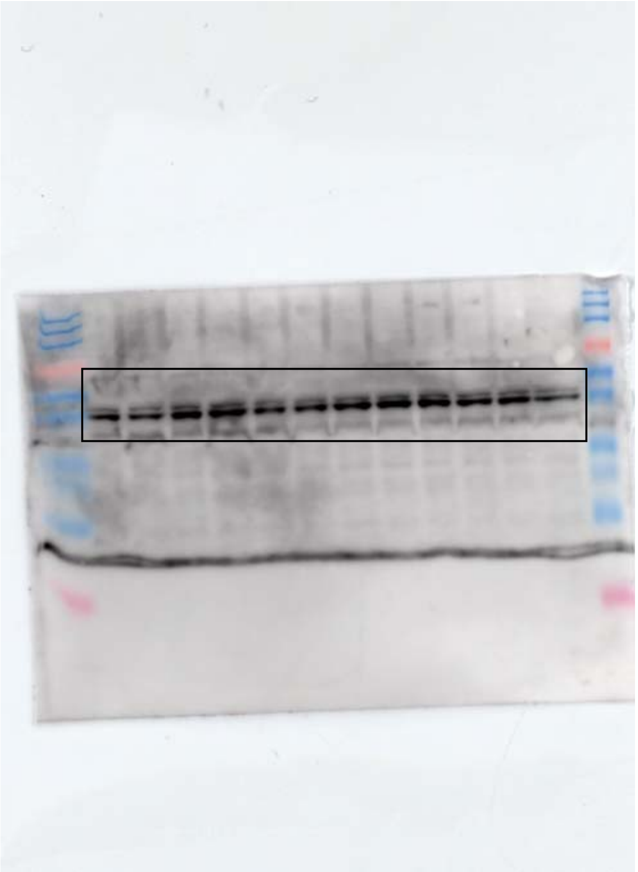

Figure 4C β-actin

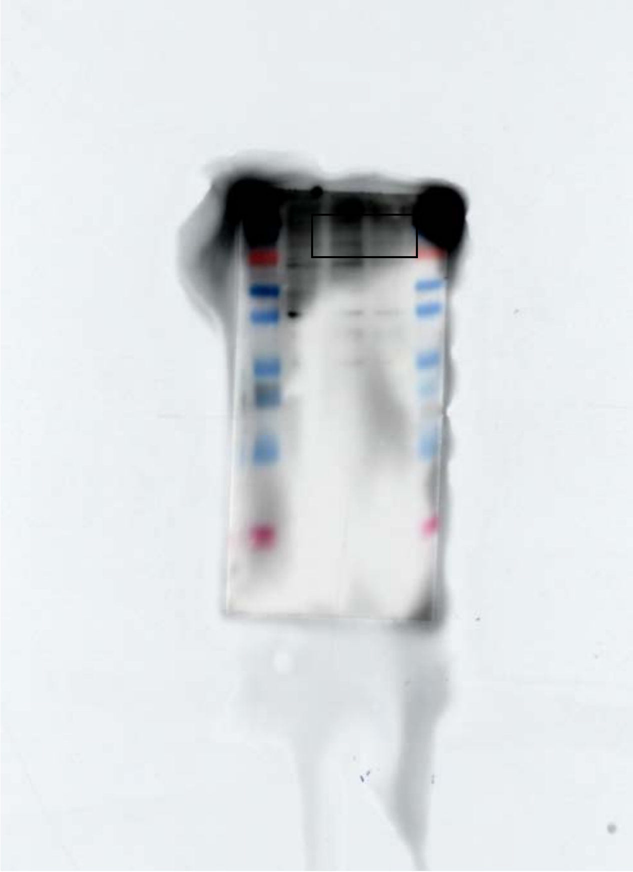

Figure 4F HMGCR

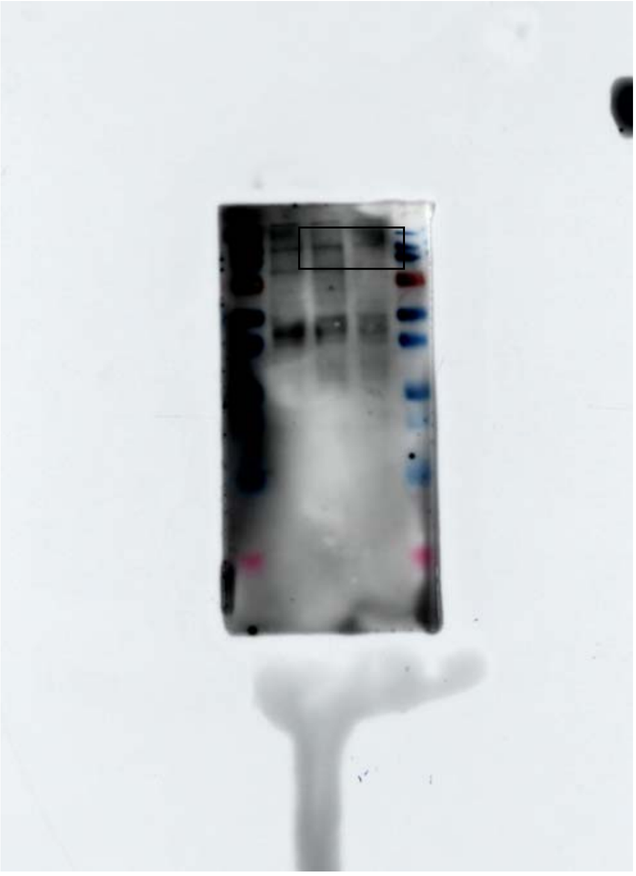

Figure 4F SREBP2

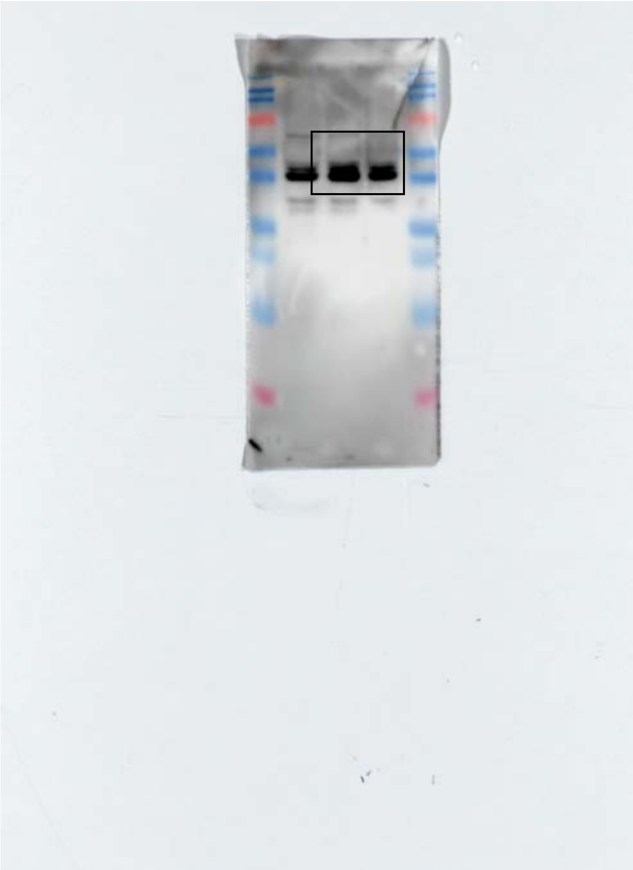

Figure 4F β-actin

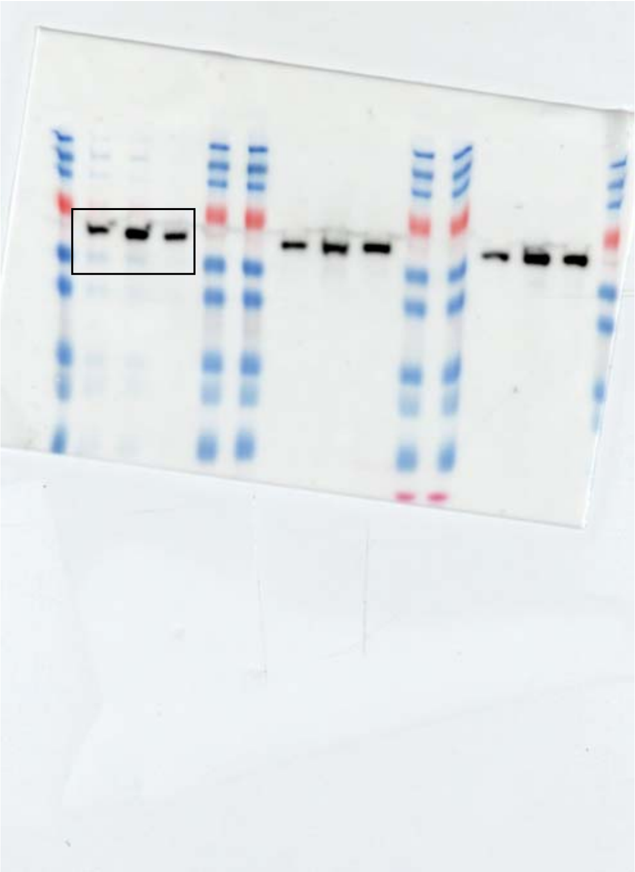

Figure 5A SHP-1

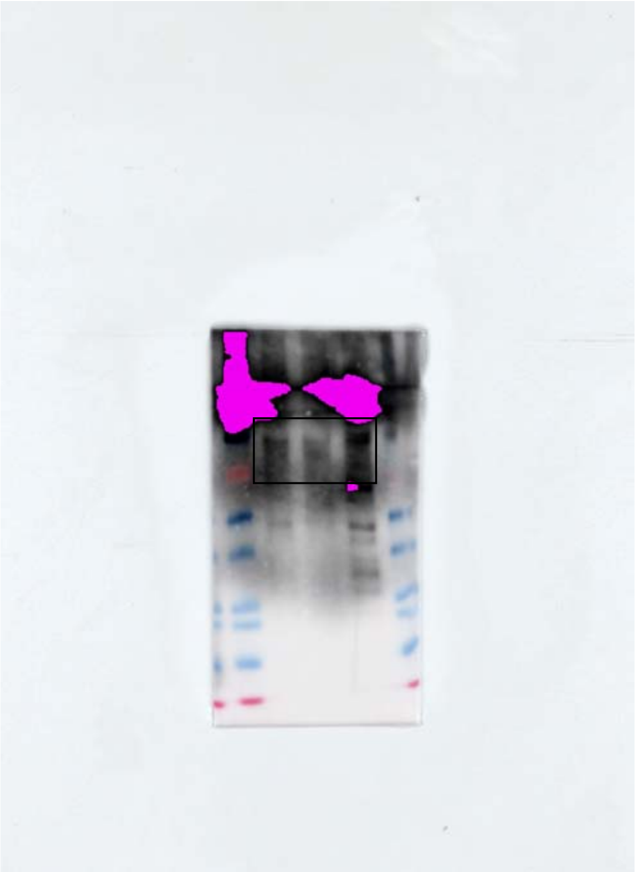

Figure 5A p-STAT6

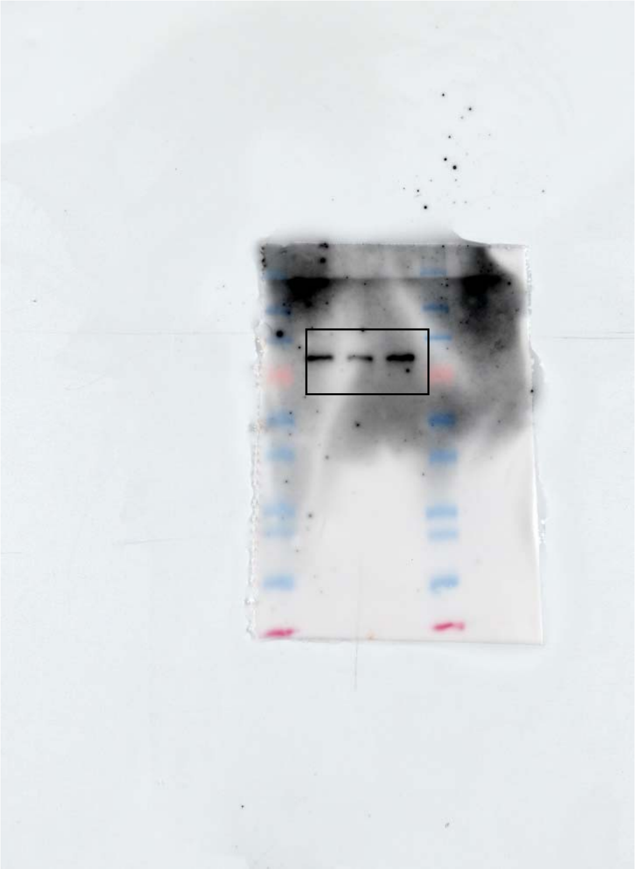
Figure 5A STAT6

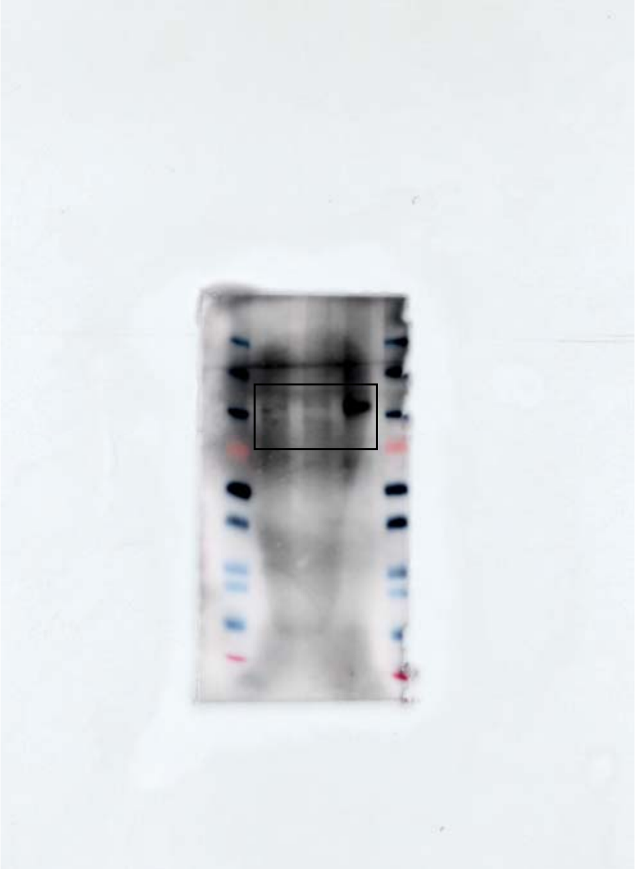
Figure 5A p-JAK1

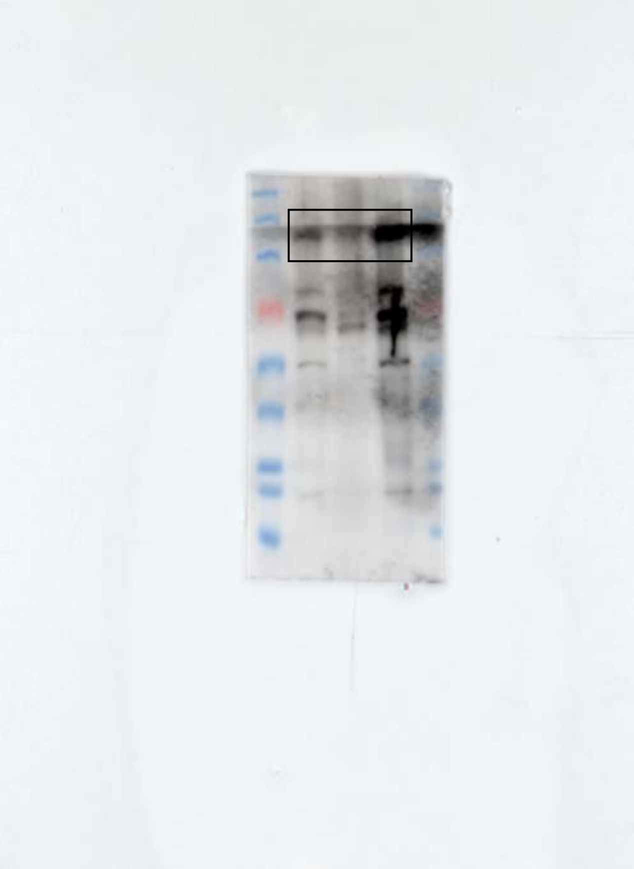

Figure 5A JAK1

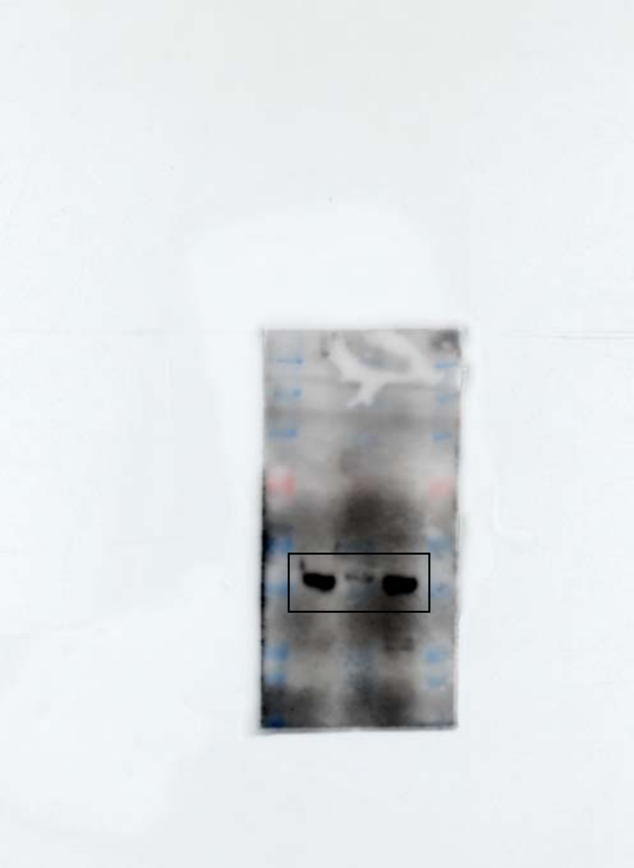

Figure 5A Arg1

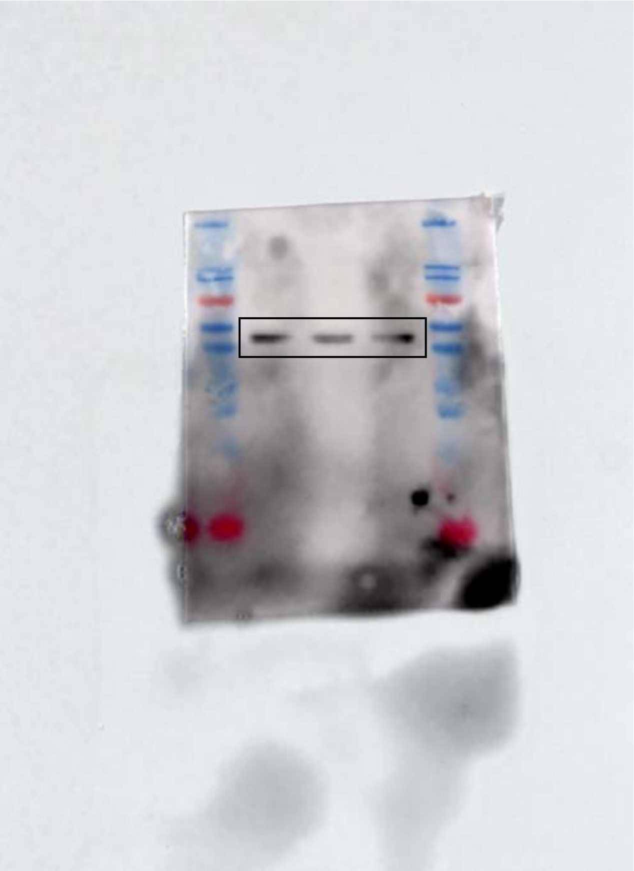

Figure 5A β-actin

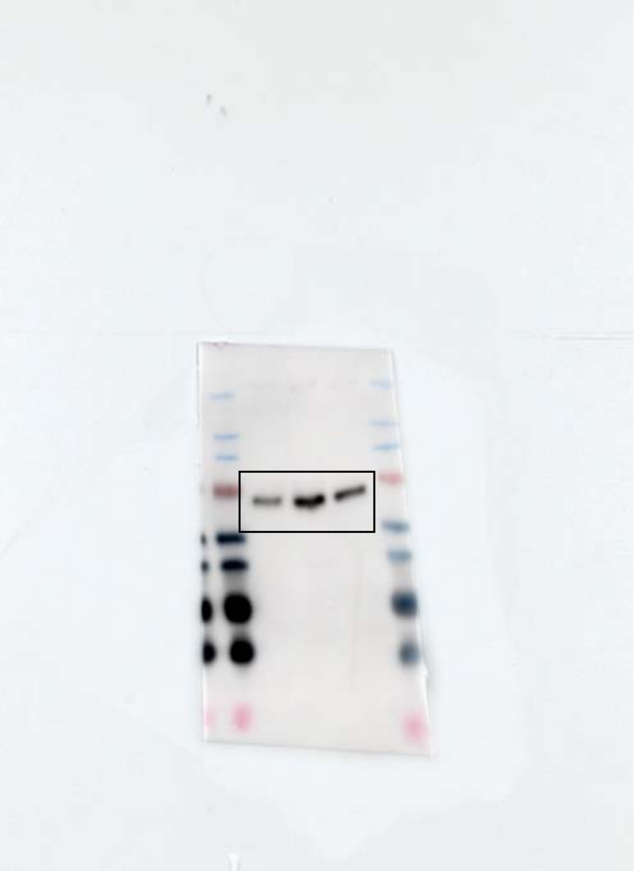

Figure 5B SHP-1

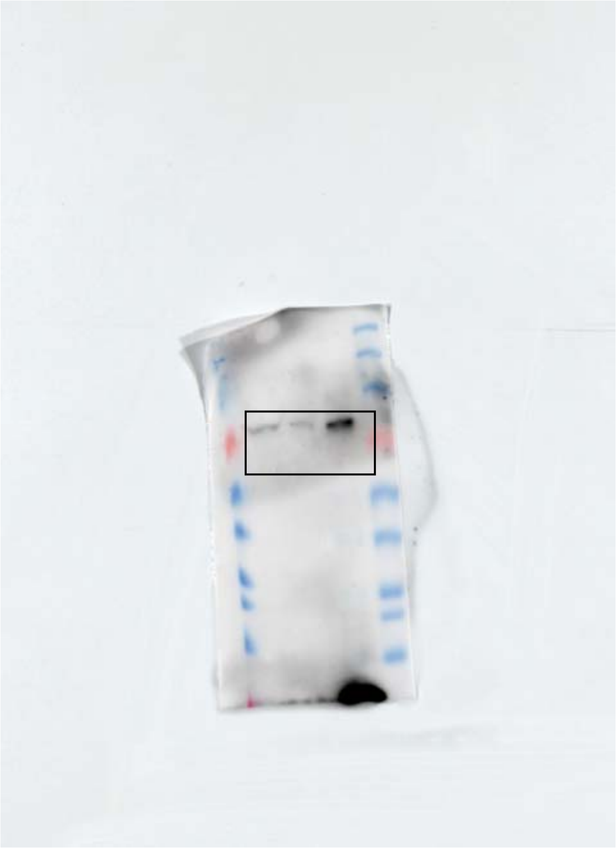

Figure 5B p-STAT6

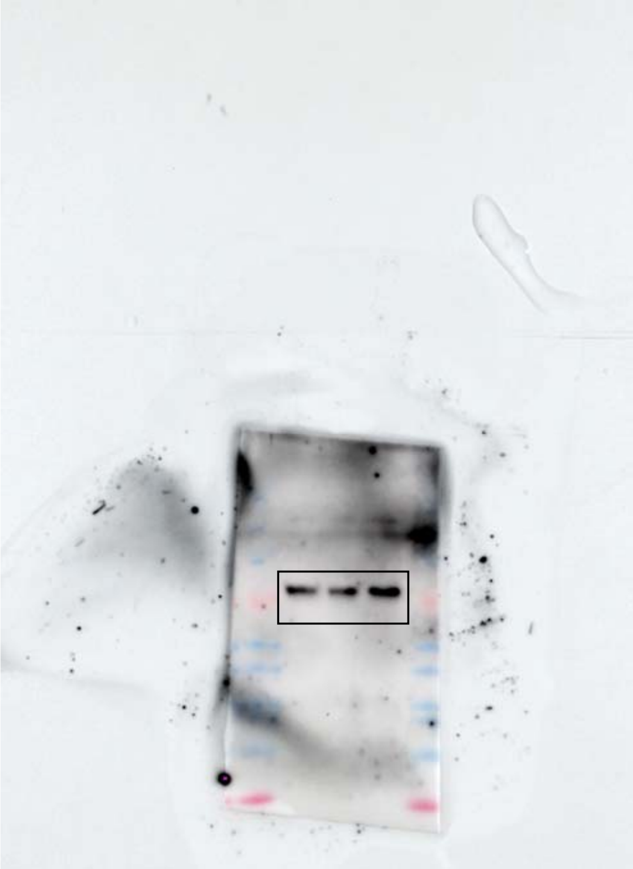

Figure 5B STAT6

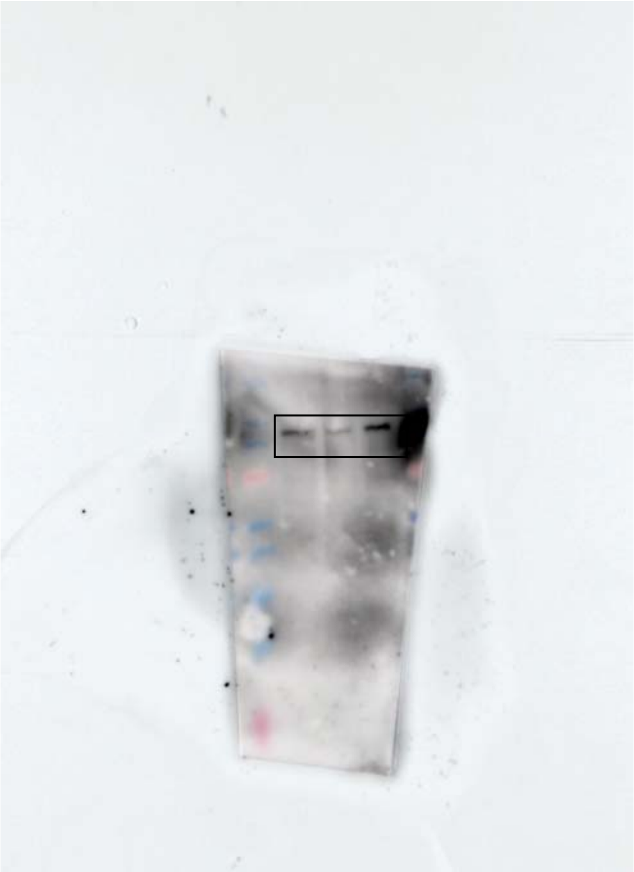

Figure 5B p-JAK1

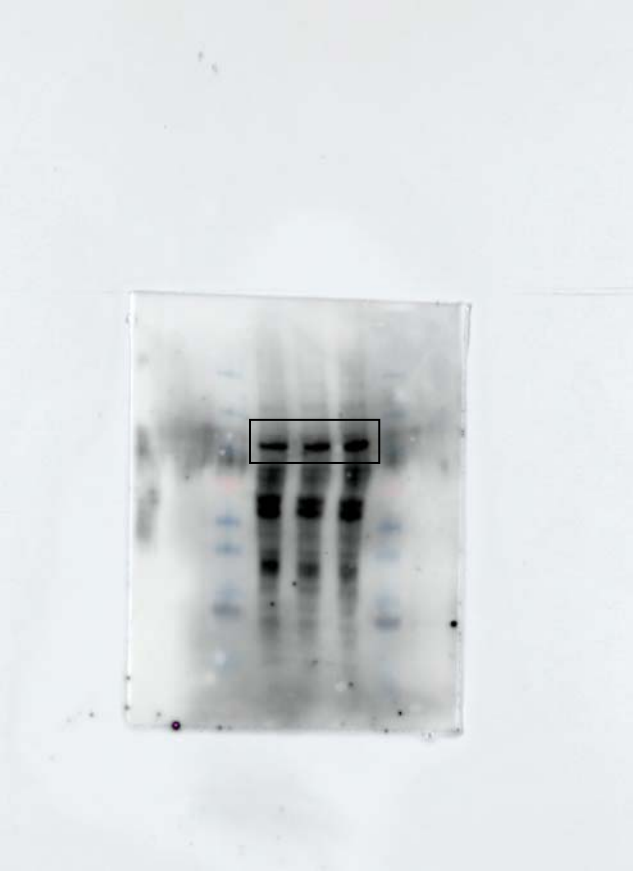

Figure 5B JAK1

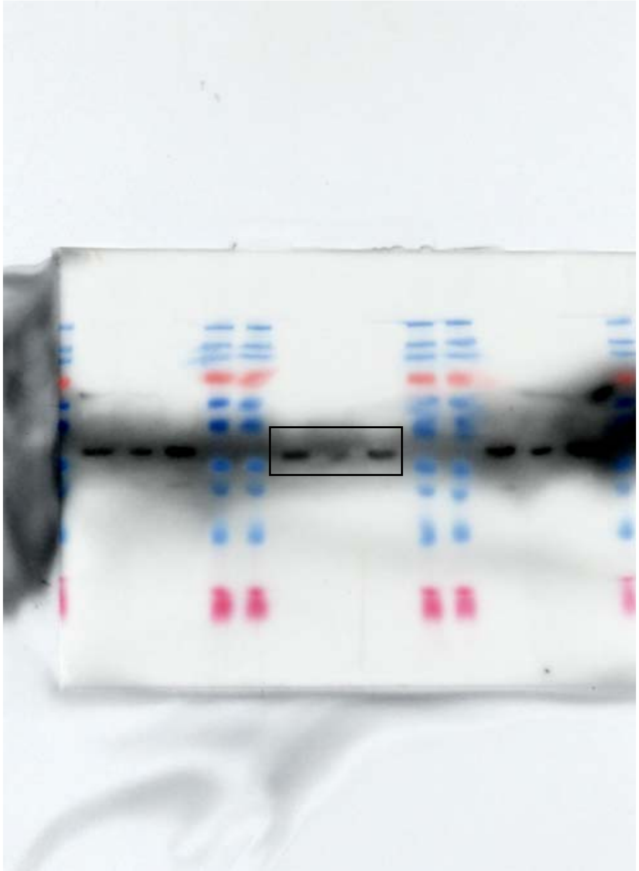

Figure 5B Arg1

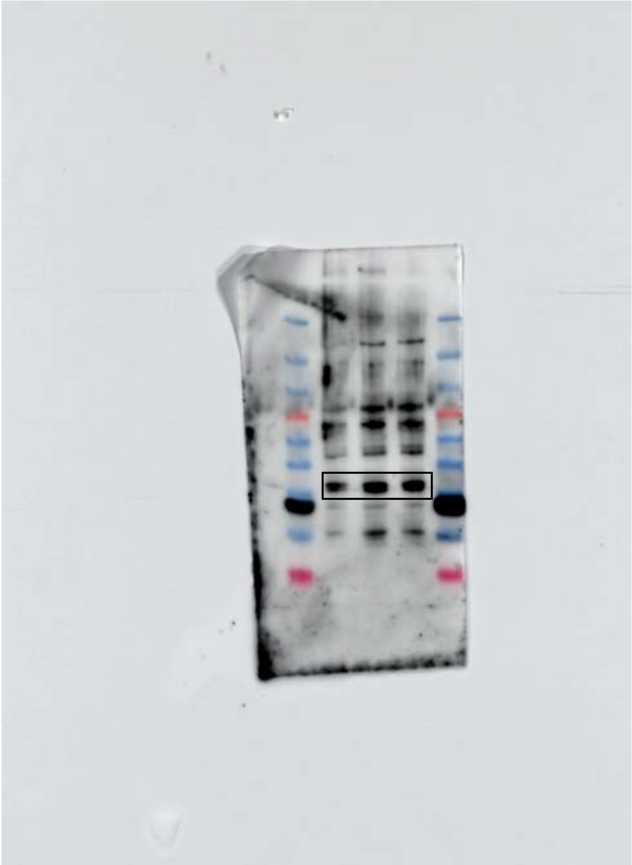

Figure 5B CD33

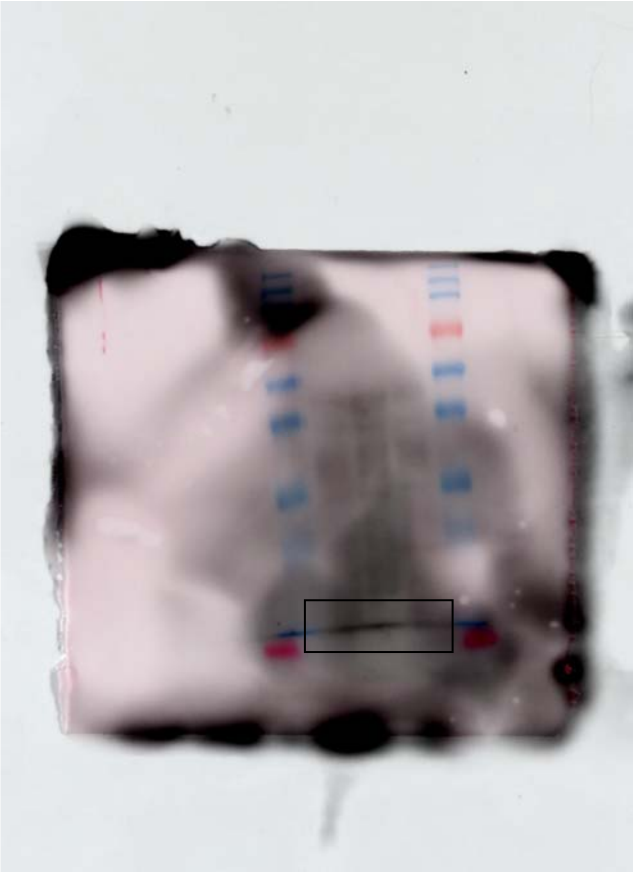

Figure 5B IL-1β

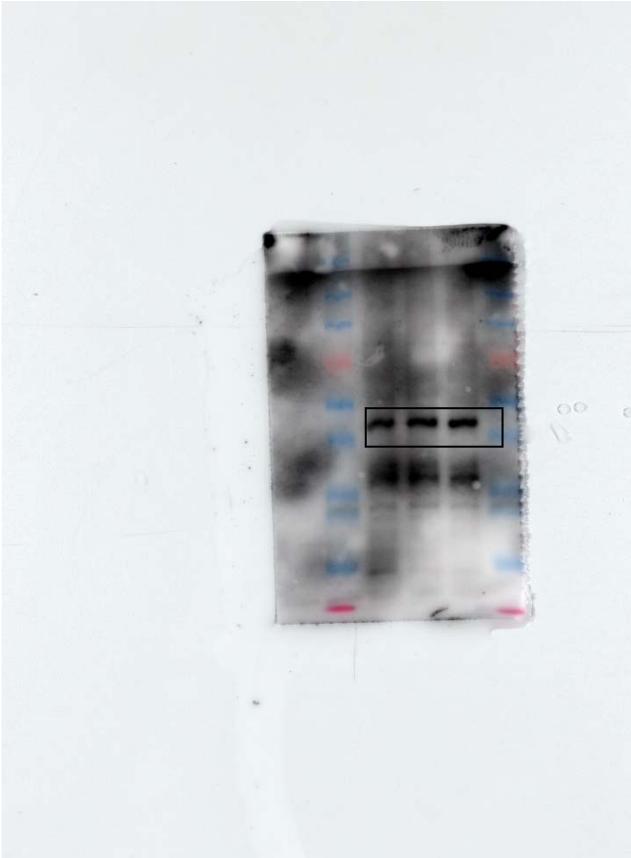

Figure 5B β-actin

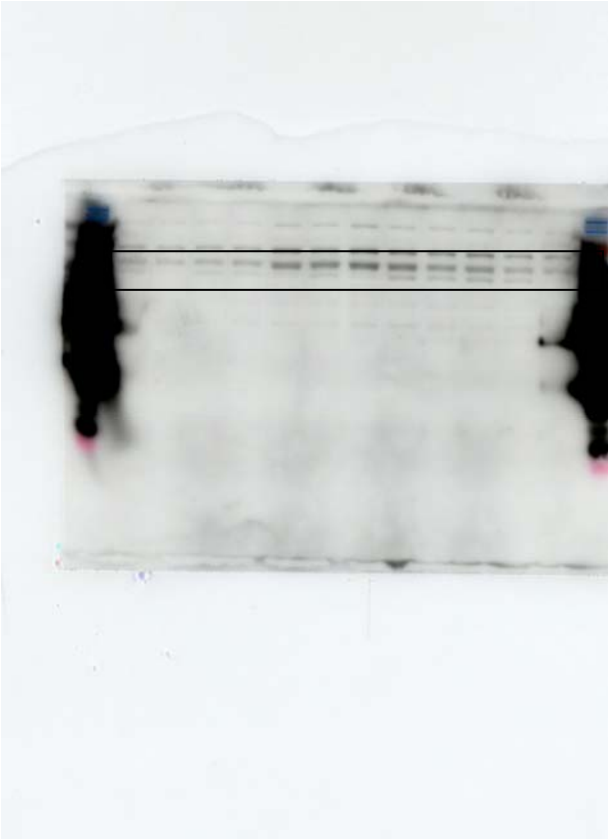
Figure 5C SHP-1

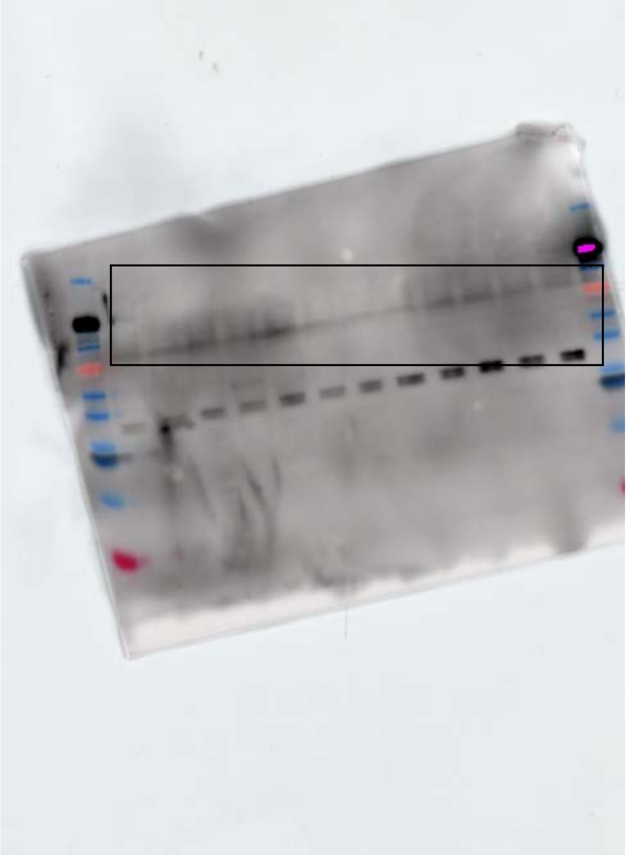
Figure 5C p-STAT6

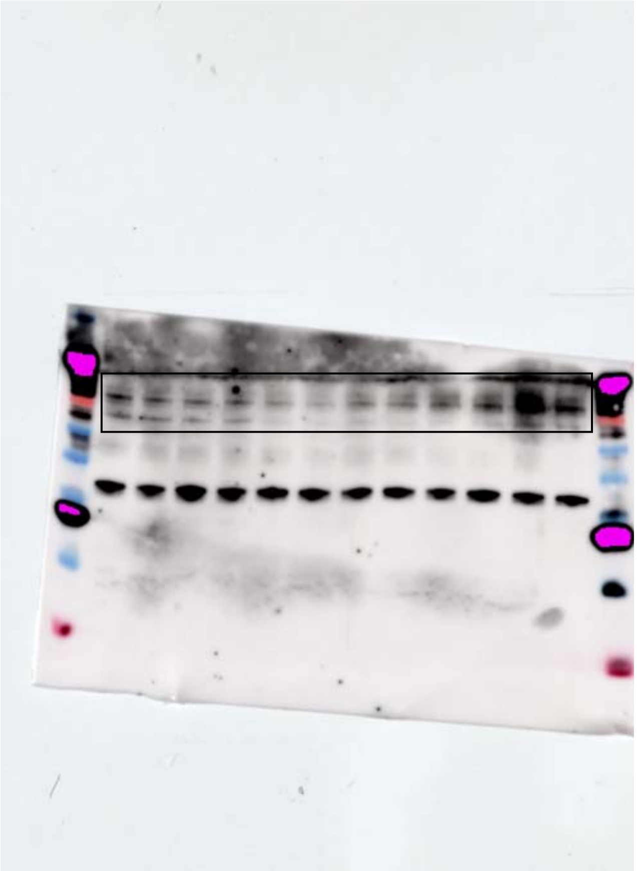

Figure 5C STAT6

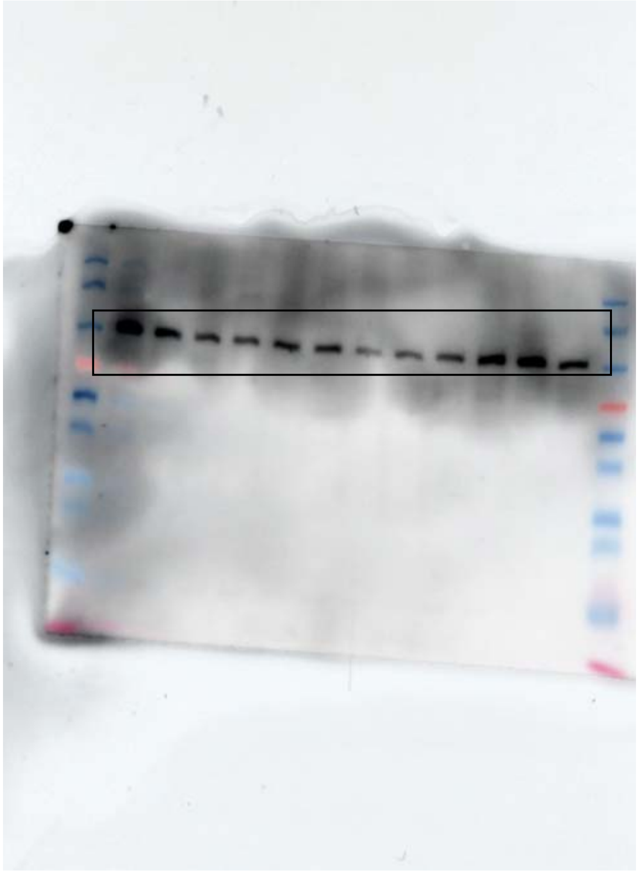

Figure 5C p-JAK1

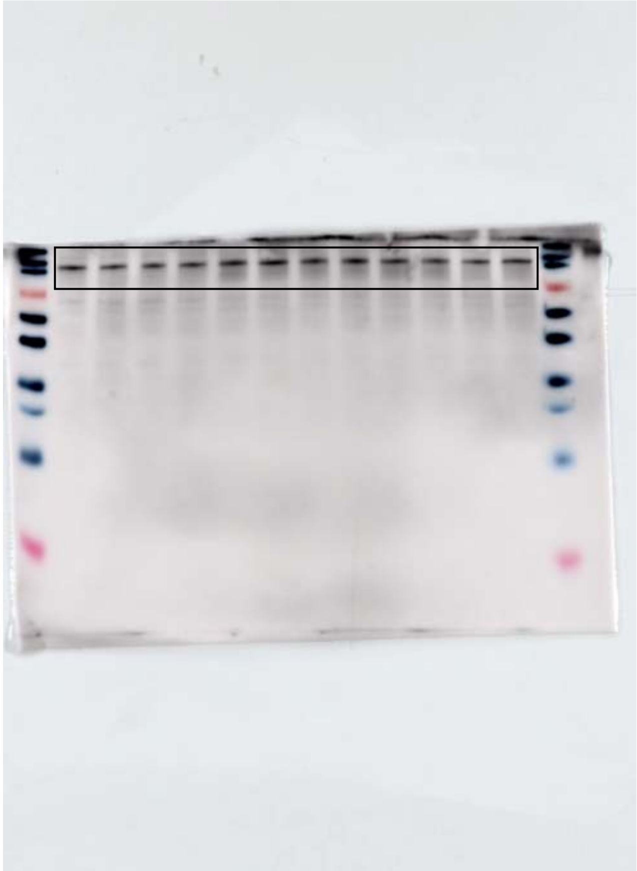

Figure 5C JAK1

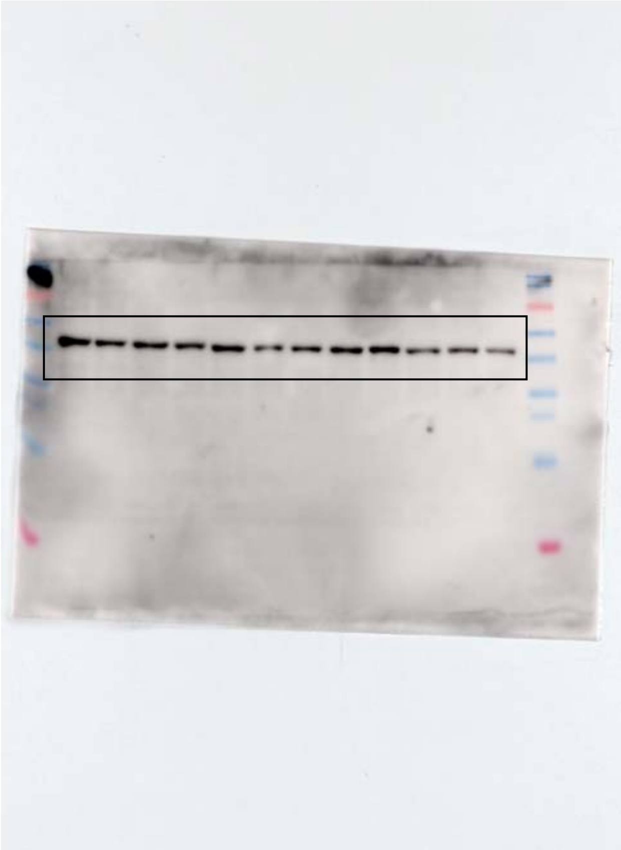

Figure 5C β-actin

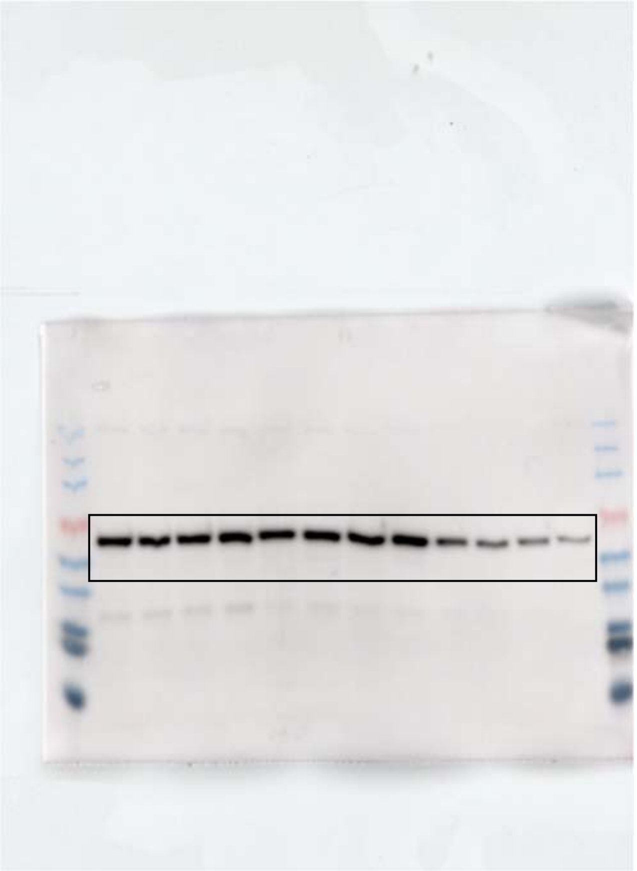

Figure 5D SHP-1

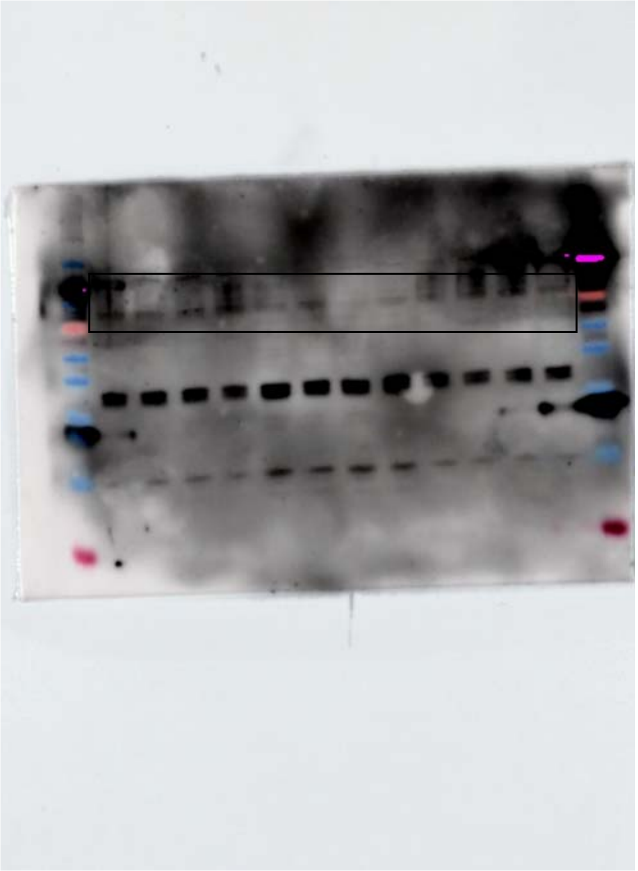

Figure 5D p-STAT6

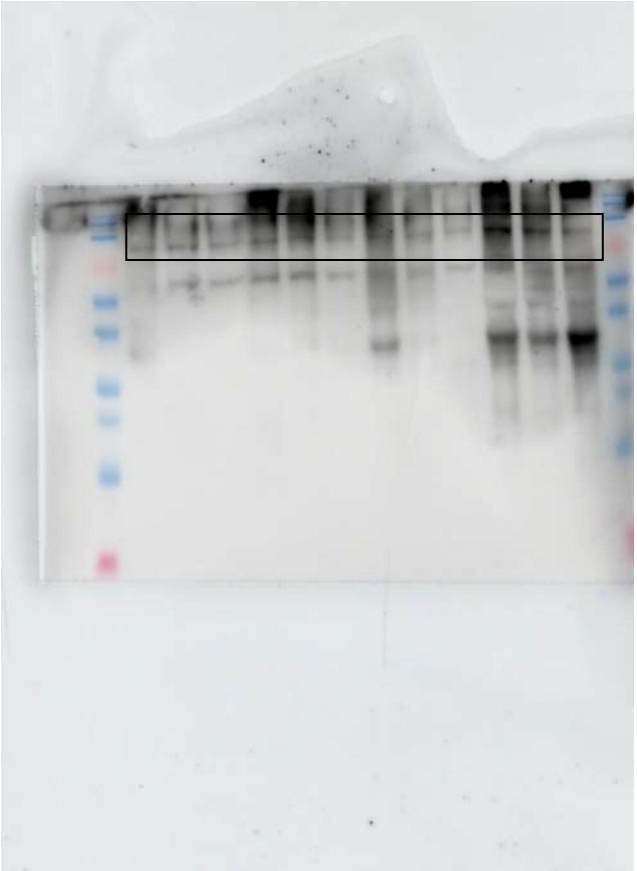

Figure 5D STAT6

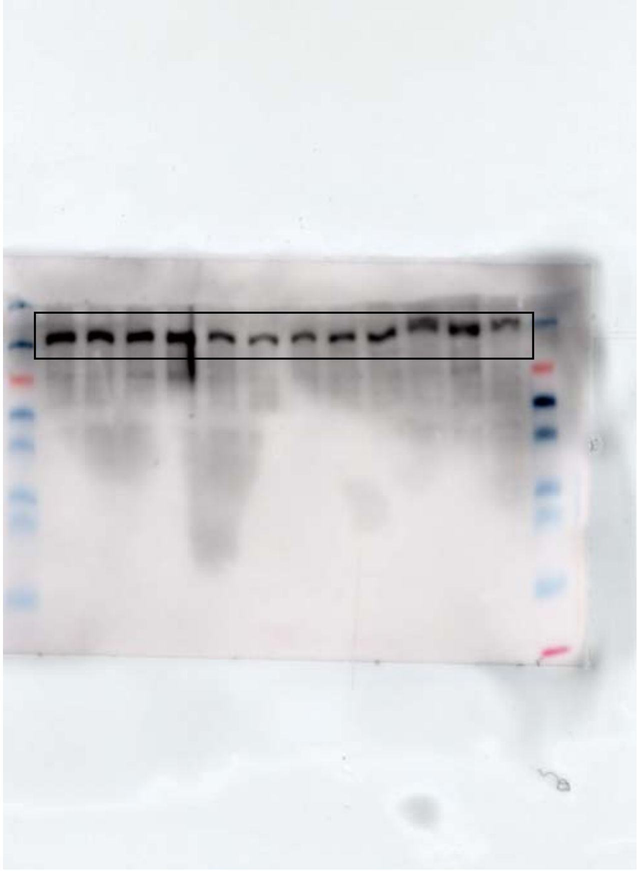

Figure 5D p-JAK1

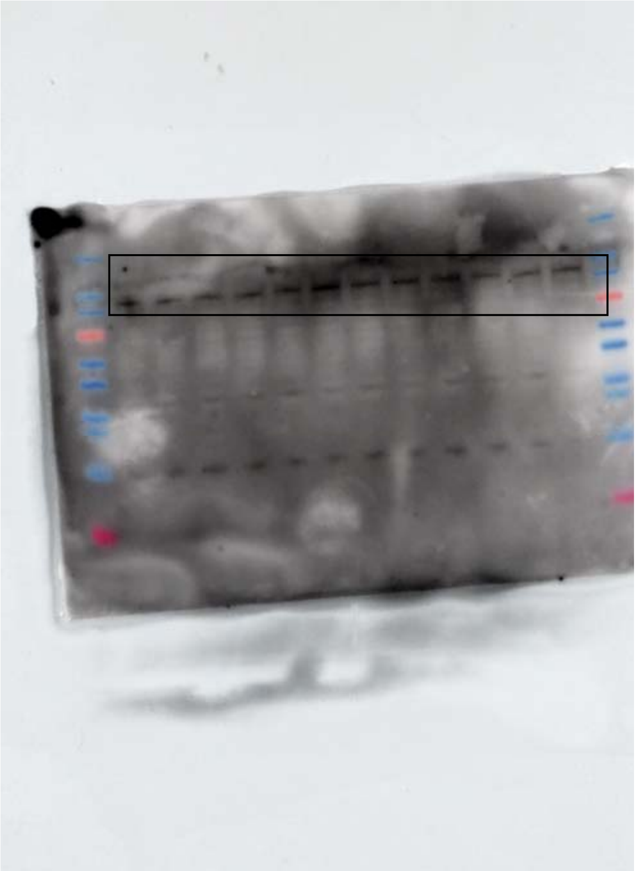

Figure 5D JAK1

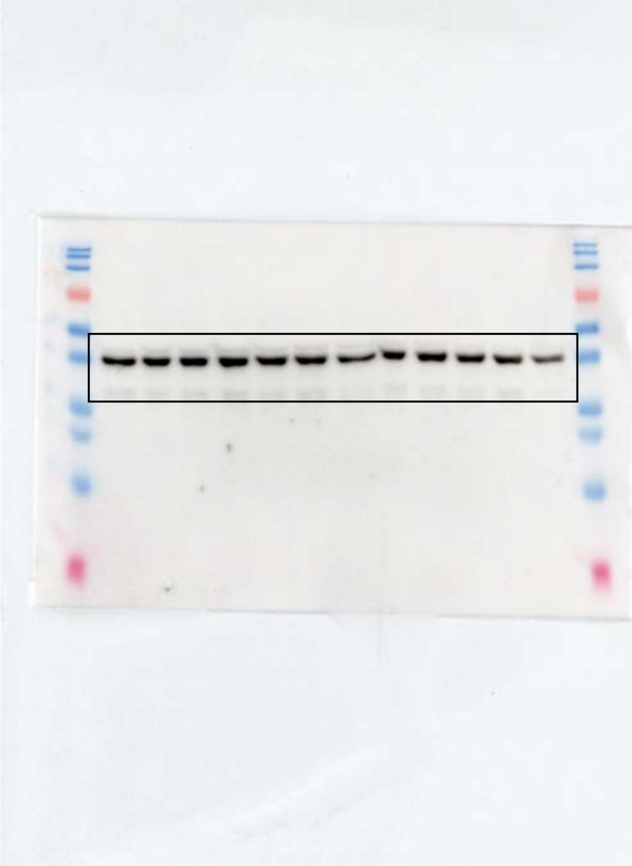

Figure 5D β-actin

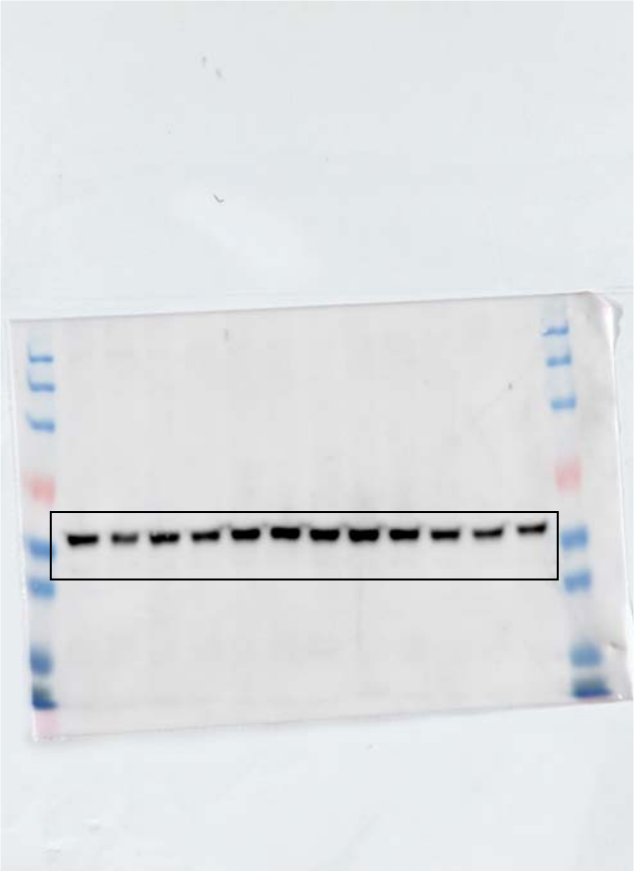
Figure 5E SHP-1

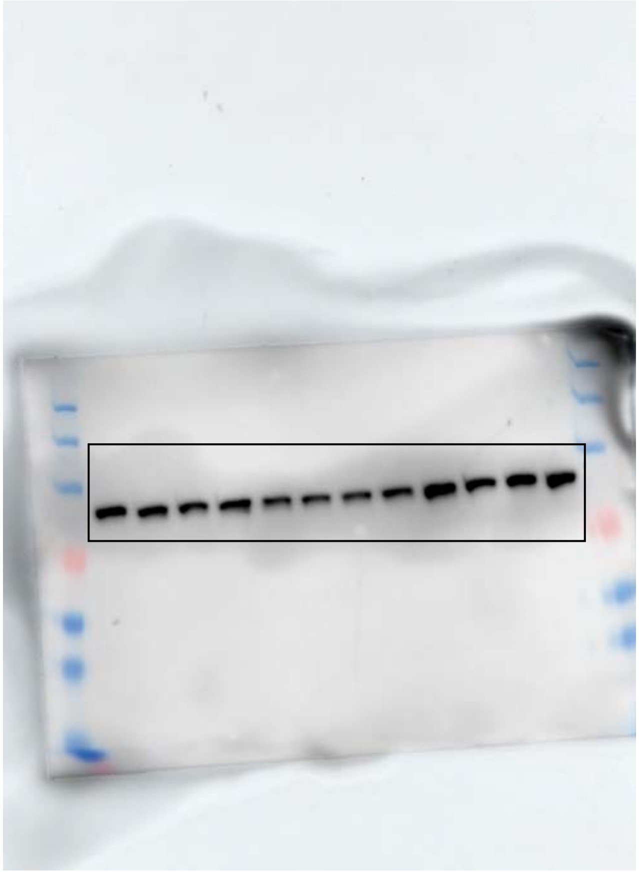
Figure 5E p-STAT6

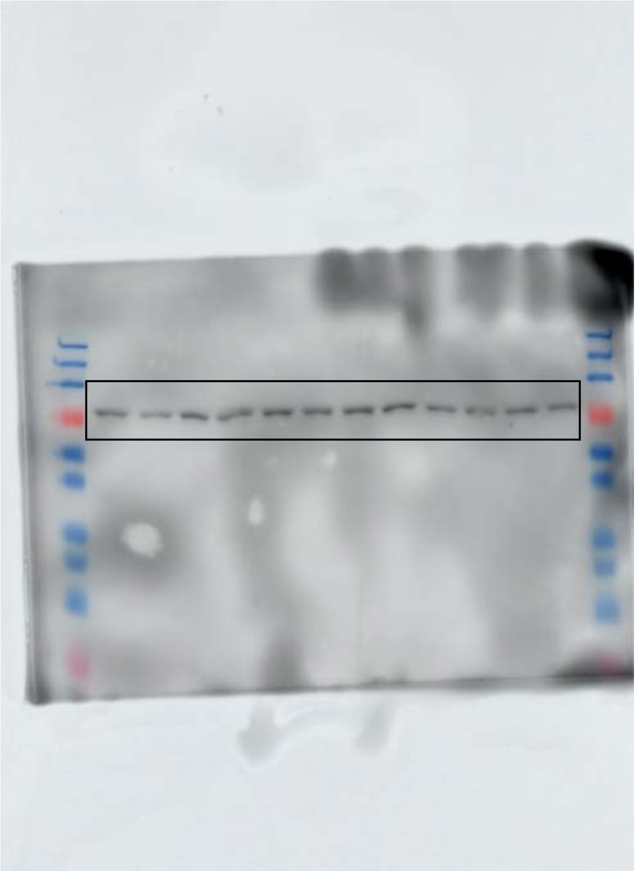

Figure 5E STAT6

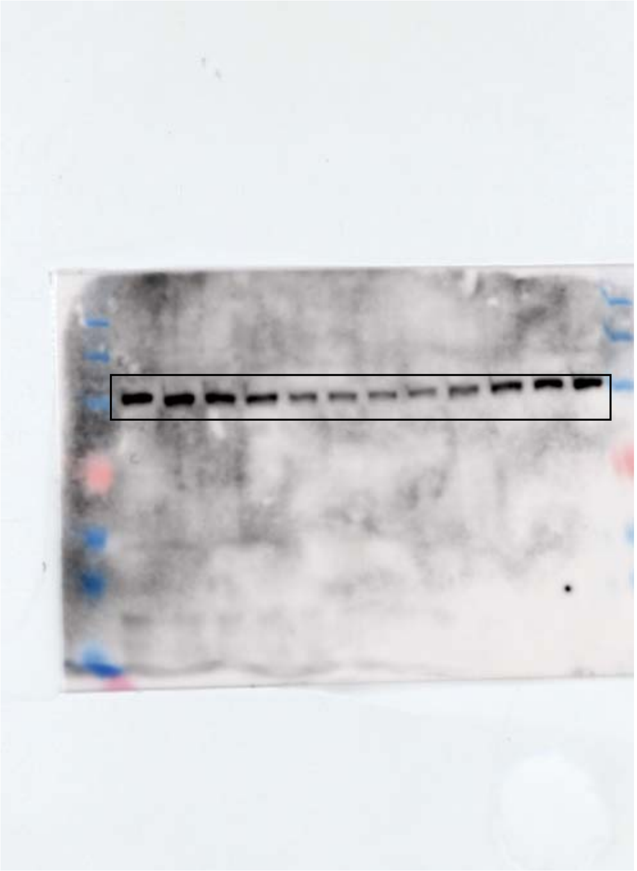

Figure 5E p-JAK1

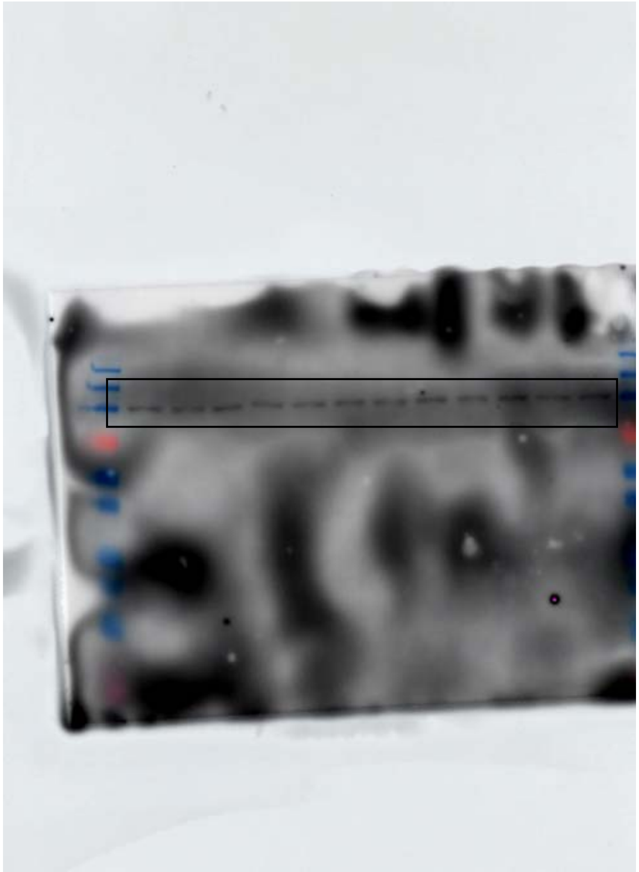

Figure 5E JAK1

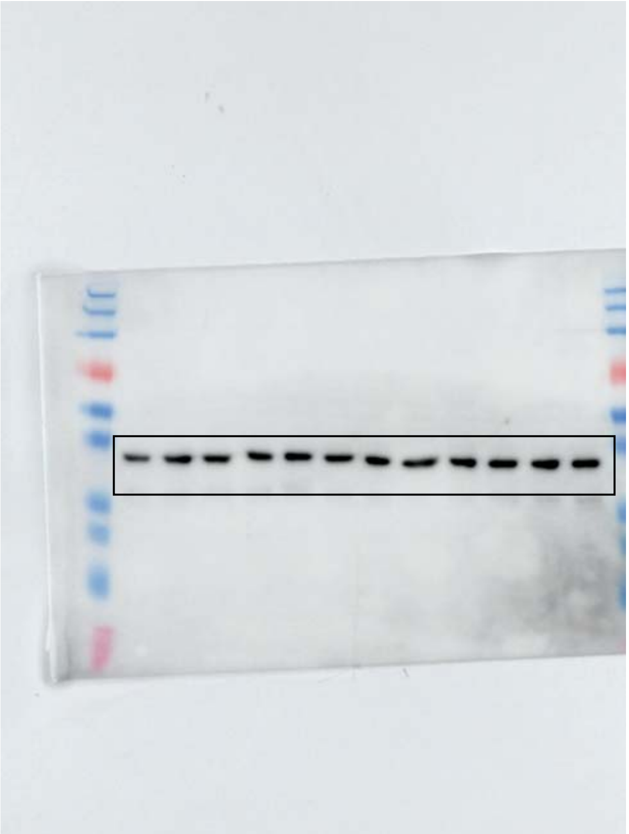

Figure 5E β-actin

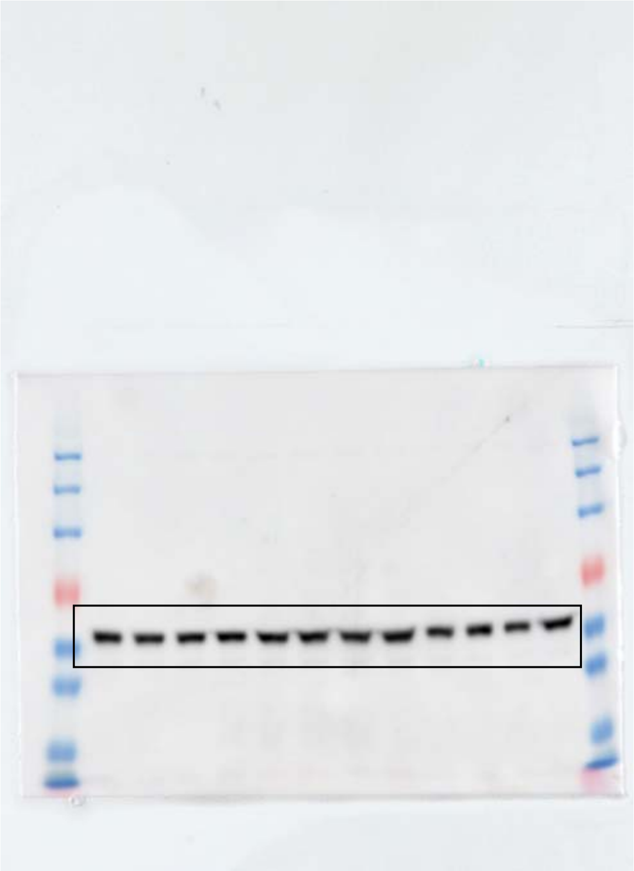

Figure 5F SHP-1

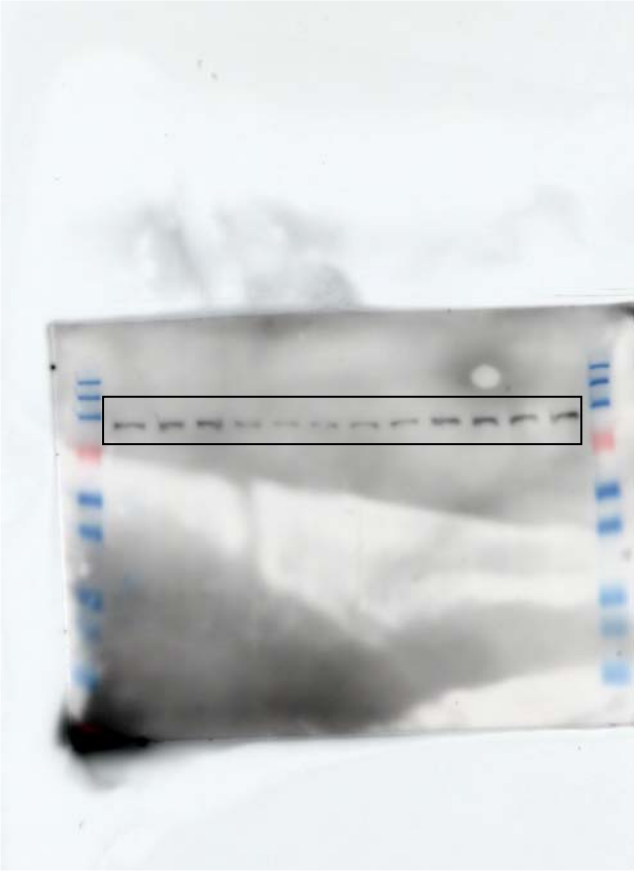

Figure 5F p-STAT6

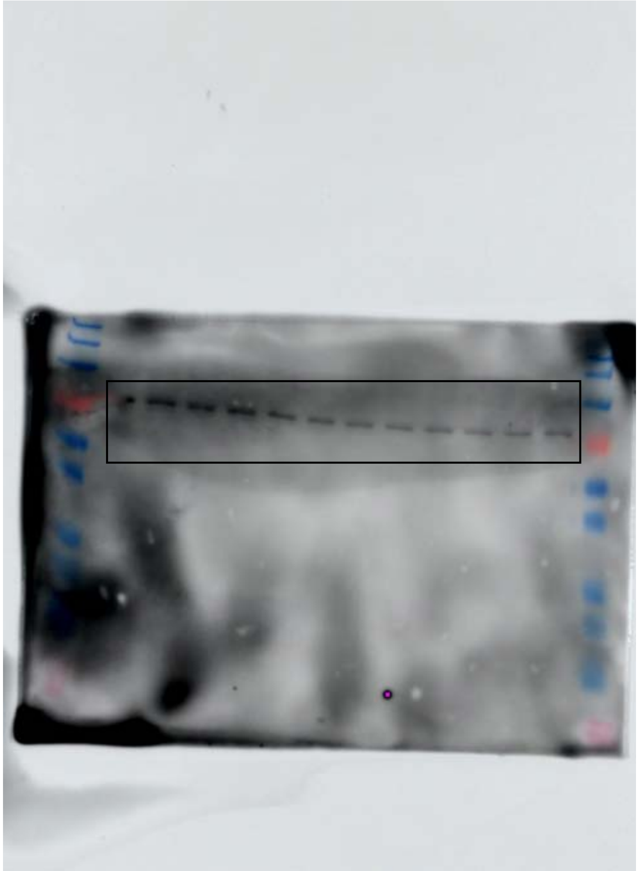

Figure 5F STAT6

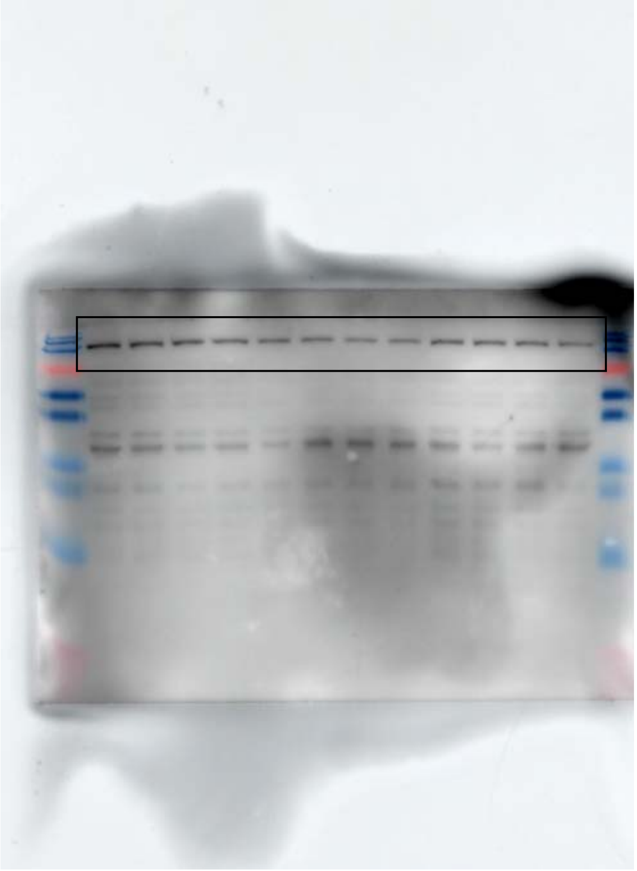

Figure 5F p-JAK1

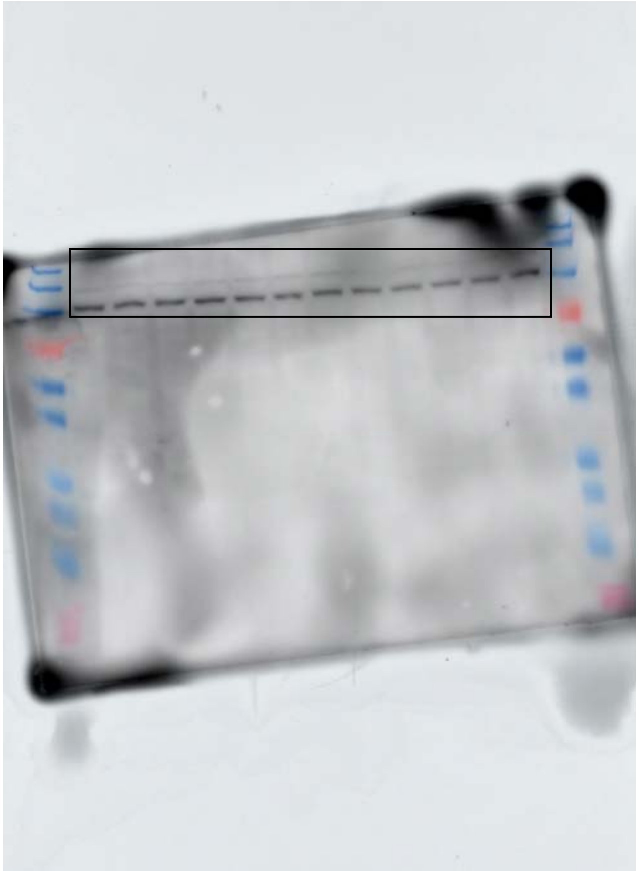

Figure 5F JAK1

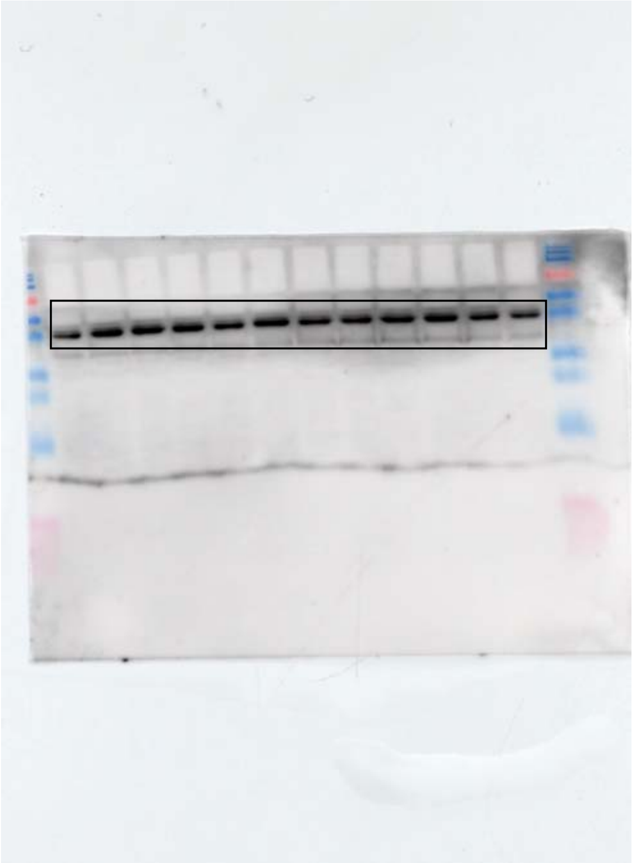

Figure 5F β-actin
